# BK channels in microglia are required for morphine-induced hyperalgesia

**DOI:** 10.1038/ncomms11697

**Published:** 2016-05-31

**Authors:** Yoshinori Hayashi, Saori Morinaga, Jing Zhang, Yasushi Satoh, Andrea L. Meredith, Takahiro Nakata, Zhou Wu, Shinichi Kohsaka, Kazuhide Inoue, Hiroshi Nakanishi

**Affiliations:** 1 Department of Aging Science and Pharmacology, Faculty of Dental Sciences, Kyushu University, Fukuoka 812-8582, Japan; 2 Department of Anesthesiology, National Defense Medical College, Tokorozawa 359-8513, Japan; 3 Department of Physiology and Program in Neuroscience, University of Maryland School of Medicine, Baltimore, Maryland 21201, USA; 4 Department of Molecular and Cellular Anatomy, Faculty of Health Promotional Sciences, Tokoha University, Hamamatsu, Shizuoka 431-2102, Japan; 5 Department of Neurochemistry, National Institute of Neuroscience, Kodaira, Tokyo 187-8502, Japan; 6 Department of Molecular and System Pharmacology, Graduate School of Pharmaceutical Sciences, Kyushu University, Fukuoka 812-8582, Japan; 7 AMED-CREST, Japan Agency for Medical Research and Development, 1-7-1, Otemachi, Chiyoda-ku, Tokyo 100-004, Japan

## Abstract

Although morphine is a gold standard medication, long-term opioid use is associated with serious side effects, such as morphine-induced hyperalgesia (MIH) and anti-nociceptive tolerance. Microglia-to-neuron signalling is critically involved in pain hypersensitivity. However, molecules that control microglial cellular state under chronic morphine treatment remain unknown. Here we show that the microglia-specific subtype of Ca^2+^-activated K^+^ (BK) channel is responsible for generation of MIH and anti-nociceptive tolerance. We find that, after chronic morphine administration, an increase in arachidonic acid levels through the μ-opioid receptors leads to the sole activation of microglial BK channels in the spinal cord. Silencing BK channel auxiliary β3 subunit significantly attenuates the generation of MIH and anti-nociceptive tolerance, and increases neurotransmission after chronic morphine administration. Therefore, microglia-specific BK channels contribute to the generation of MIH and anti-nociceptive tolerance.

## Abstract (web summary)

Long-term use of opioids can lead to a paradoxical increase in pain sensitivity. Here, Hayashi *et al*. link activation of potassium channels on microglia with morphine-induced hyperalgesia and anti-nociceptive tolerance in mice.

## Graphical abstract

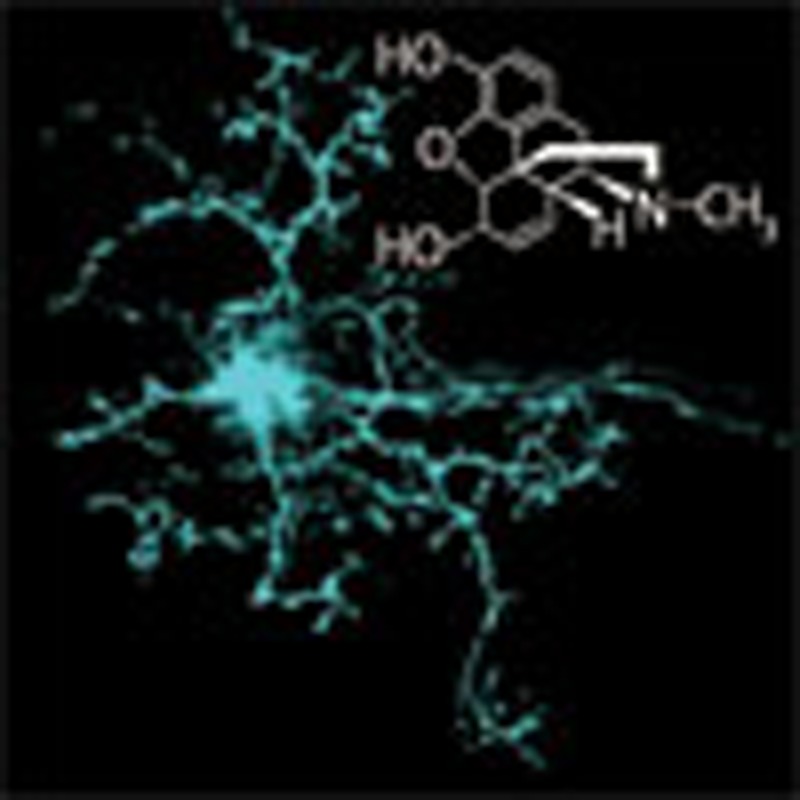

Opioids are still gold standard medications for pain management in the clinical setting, even after the discovery of novel therapeutic strategies for chronic pain. Despite their common use, there are still serious problems associated with the use of opioids, including opioid-induced hyperalgesia and anti-nociceptive tolerance, which frequently hamper medical adherence. Hyperalgesia is defined as a state of nociceptive sensitization. The long-term opioid therapy in patients for chronic pain paradoxically increases pain sensitivity. Anti-nociceptive tolerance is characterized as a progressive reduction of analgesic effect with an equivalent dose of opioids. In such cases, clinicians perform dose escalation to provide effective pain management, however, this increases the incidence and risk of adverse side effects, such as respiratory depression, nausea, sedation, euphoria or itching^1^. A better comprehension of opioid-induced hyperalgesia and anti-nociceptive tolerance might facilitate the development of a novel strategy for long-term opioid use, which could reduce the undesirable adverse side effects resulting from dose escalation.

NMDA receptors (NMDARs) are well-studied, and are known to be associated with opioid-induced hyperalgesia and anti-nociceptive tolerance^2^,^3^. In addition, treatments targeting their downstream molecules^4^,^5^,^6^ are effective for opioid-induced hyperalgesia and anti-nociceptive tolerance. Blockade of NMDARs effectively inhibits the generation of opioid-induced hyperalgesia and anti-nociceptive tolerance, but it can lead to disturbed physiological brain function, including hallucinations, dizziness, nightmares and headache. Effective medications with fewer potential side effects are desired for long-term opioid use. An extremely low concentration of ketamine, a classic analgesic drug, can alleviate the generation of opioid-induced hyperalgesia and anti-nociceptive tolerance in the clinical setting^7^,^8^,^9^ or in the animal model^10^,^11^,^12^, even though this concentration of ketamine itself has no analgesic effect^13^,^14^. The mechanism underlying the improvement of opioid-induced hyperalgesia and anti-nociceptive tolerance with an extremely low concentration of ketamine remains poorly understood.

Ketamine has a chiral centre in its structure, and the enantiomers have different analgesic potency and/or incidence rates of side effects^15^. Intriguingly, ketamine has several molecular targets^16^,^17^. We have identified that large conductance Ca^2+^-activated K^+^ (BK) channels in microglia represent a potential novel molecular target for *S*-ketamine^18^. Accumulating evidence has indicated that opioid-induced hyperalgesia and anti-nociceptive tolerance are associated with microglial activation, including activation of P2X4 receptors (P2X4Rs)^19^,^20^ or bioactive sphingolipids^21^.

In this study, we have examined the possible involvement of microglial BK channels in the generation of opioid-induced hyperalgesia and anti-nociceptive tolerance. We herein show that BK channels on microglia are responsible for the generation of opioid-induced hyperalgesia and anti-nociceptive tolerance. Pharmacological blockade or the genetic deletion of BK channels in microglia prevented morphine-induced hyperalgesia (MIH) and anti-nociceptive tolerance. Increased level of arachidonic acid (AA) or its metabolites through μ-opioid receptors directly activated BK channels in microglia following chronic morphine administration. Inhibition of BK channels attenuated the membrane translocation of P2X4Rs in microglia. BK channels in microglia modulated the Ca^2+^ influx through store-operated Ca^2+^ entry (SOCE), which promoted the secretion of brain-derived neurotrophic factor (BDNF) after morphine stimulation. Therefore, BK channels in microglia play a crucial role in the ionic homeostasis that regulates the cellular activation state, resulting in an increase in the pain perception and attenuation of opioid anti-nociception.

## Results

### A BK channel inhibitor attenuates MIH

The accumulating evidence suggests an involvement of a similar molecular mechanism underlying the neuropathic pain in a paradoxically worsening of pain following the long-term use of morphine^19^,^20^. To address whether microglial BK channels were activated after chronic morphine treatment, we conducted patch clamp recordings using GFP-labelled microglial cells in the spinal cord slice preparations from *Iba1*^*+/GFP*^ mice that had been treated with morphine (10 mg kg^−1^, i.p., twice a day for 5 consecutive days). The robust activation of outward currents was observed in the lamina I microglia of mice after a 5-day treatment with morphine compared with saline-treated mice (Supplementary Fig. 1a,b). The outward rectifier currents recorded in the lamina I microglia from the morphine-treated mice were sensitive to bath application of IbTx, a BK channel-specific inhibitor, but insensitive to apamin, a SK channel inhibitor (Supplementary Fig. 1a,b). On the other hand, BK currents recorded from the lamina I neurons or astrocytes in the spinal cord did not show obvious activation following morphine administration (Supplementary Fig. 2).

**Figure 1:**
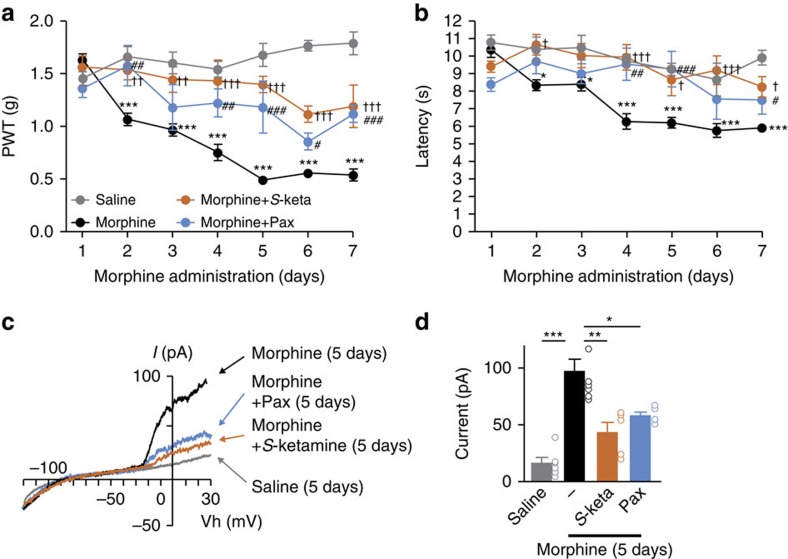
Attenuation of morphine-induced hyperalgesia by supplementation with *S*-ketamine or paxilline. (**a**,**b**) The PWT (paw withdrawal threshold; mechanical stimulation) (**a**) or hot plate latency (thermal stimulation) (**b**) following systemic administration of morphine (10 mg kg^−1^). *S*-ketamine (2 mg kg^−1^) or paxilline (2.2 μg kg^−1^) were administered 30 min prior to morphine administration. Saline (*n*=8), Morphine (*n*=15), Morphine+*S*-ketamine (*n*=6), Morphine+Paxilline (*n*=5). (**c**) Typical traces of BK currents in the lamina I spinal microglia after 5 days of morphine administration with or without *S*-ketamine or paxilline. (**d**) The amplitude of BK currents at +30 mV (*n*=6/8/5/5 from three to four mice). The data represent the means±s.e.m. ***P=0.0001, 0.0001, 0.0001, 0.0001, 0.0001, 0.0001 (Saline versus Morphine), ^††^*P*=0.0088, 0.0065, ^†††^*P*=0.0001, 0.0001, 0.0006, 0.0001 (Morphine versus *S*-ketamine), ^#^*P*=0.0321, ^##^*P*=0.0070, 0.0091, ^###^*P*=0.0001, 0.0010 (Morphine versus Paxilline), (drug × time point interaction): F_3, 180_=125.24, a two-way ANOVA followed by the Bonferroni *post hoc* test (**a**). *P=0.0125, 0.0103, ***P=0.0001, 0.0001, 0.000, 0.0001 (Saline versus Morphine), ^†^*P*=0.0313, 0.0467, 0.0491, ^†††^*P*=0.0001, 0.0003 (Morphine versus *S*-ketamine), ^#^*P*=0.0145, ^##^*P*=0.0028, ^###^*P*=0.0076 (Morphine versus Paxilline) (drug × time point interaction): F_3, 180_=49.80, a two-way ANOVA followed by the Bonferroni *post hoc* test (**b**). *P=0.0197, **P=0.0013, ***P=0.0001 F_2, 20_=17.68, a one-way ANOVA followed by the Tukey's *post hoc* test (**d**).

Based on our previous finding that BK channels in the spinal microglia were a potential novel molecular target of *S*-ketamine^18^, we considered that the effect of an ultra-low dose of *S*-ketamine on MIH and anti-nociceptive tolerance in the clinical setting might be due to suppression of the microglial BK channel activation. To address this possibility, we first assessed the effects of ketamine enantiomers on pain behaviour^15^. Supplementation with *S*-ketamine (2 mg kg^−1^, i.p.) significantly attenuated the generation of MIH (Fig. 1a,b) and anti-nociceptive tolerance in mice (Supplementary Fig. 3a), similar to the finding in clinical studies^7^,^8^,^9^. Preferential inhibition of anti-nociceptive tolerance by *S*-ketamine (2 mg kg^−1^, i.p.), whose concentration was not sufficient to induce analgesia, was also observed (Supplementary Fig. 3a). We next examined morphological changes in the lamina I microglia by intracellular Lucifer Yellow staining in weakly fixative-fixed spinal cord slice preparations. The mean total length of microglial processes determined by a three-dimensional (3D) measurement was significantly reduced after a 5-day treatment with morphine, whereas *S*-ketamine supplementation (2 mg kg^−1^, i.p.) inhibited these morphological alterations (Supplementary Fig. 4) in parallel with its analgesic effect. To examine a possible involvement of microglia in the generation of MIH and tolerance, we utilized minocycline, an inhibitor of microglial activation. Minocycline (20 mg kg^−1^) clearly attenuated the generation of MIH and anti-nociceptive tolerance (Supplementary Fig. 5) in a similar manner to previous reports^22^,^23^,^24^, indicating that microglia play a significant role in the generation of MIH and anti-nociceptive tolerance.

Then, we next assessed the involvement of BK channels in the generation of MIH and anti-nociceptive tolerance. Intrathecal (i.t.) administration of IbTx, but not apamin, markedly attenuated tolerance (Supplementary Fig. 3b). On the other hand, i.t. injection of NS1619, a BK channel opener, reduced the response to morphine. Paxilline, an indole diterpene from fungi which potentially and selectively inhibits BK channels, is known to cross the blood–brain barrier. Low dose of systemic administration of paxilline (2.2 μg kg^−1^) improves abnormal seizure activity without affecting motor activity^25^,^26^. These facts led us to analyse the effects of the systemic effects of paxilline on pain behaviours.

We reproduced the effects of low dose of paxilline (2.2 and 4.4 μg kg^−1^) on motor function (Supplementary Fig. 3c). Interestingly, paxilline (2.2 μg kg^−1^, i.p.) attenuated the generation of MIH and anti-nociceptive tolerance (Fig. 1a,b and Supplementary Fig. 3c), as well as the activation of BK currents in the lamina I microglia (Fig. 1c,d). Moreover, we found a significant reduction of BK currents in the lamina I microglia after the supplementation with a small dose of *S*-ketamine (2 mg kg^−1^) (Fig. 1c,d). These results suggested that microglial BK channels, a potential molecular target of low dose of ketamine, are involved in the generation of MIH and anti-nociceptive tolerance.

### Microglial BK channel activation aggravates MIH

**Figure 2:**
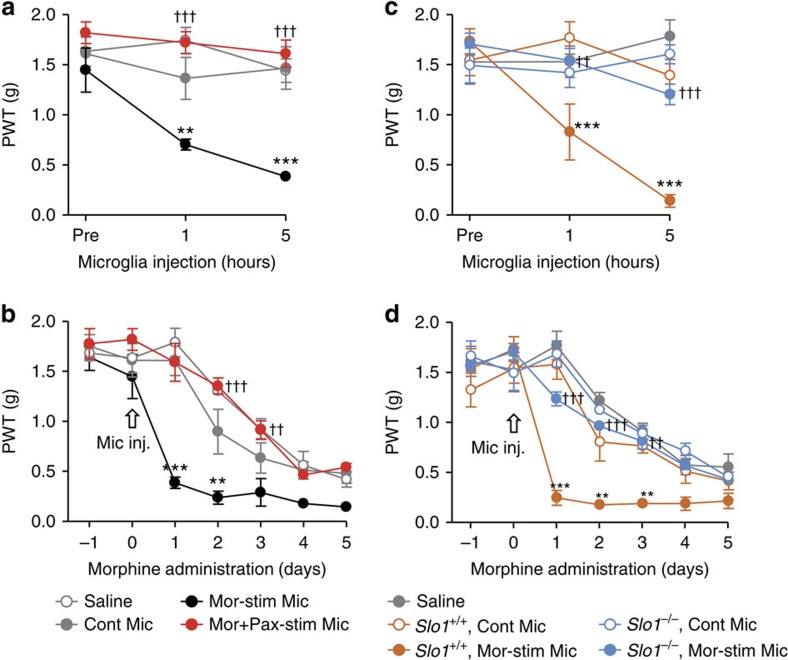
BK channels are required for the development of morphine-induced hyperalgesia. (**a**,**c**) The time course of the PWT following the i.t. injection of morphine (10 μM)-stimulated primary microglia, whose BK channels were pharmacologically (**a**) or genetically (**c**) silenced. Cont mic, non-stimulated primary microglia; Mor-stim mic, morphine-stimulated primary microglia; Mor+Pax-stim mic, morphine+paxilline (2 μM)-stimulated primary microglia (*n*=6 each). (**b**,**d**) Effects of i.t. injection of morphine-stimulated primary microglia on the development of morphine-induced hyperalgesia (*n*=6 each). The open arrow indicates the i.t. injection of primary microglia. The data represent the means±s.e.m. **P=0.0038, **P=0.0002 (Saline versus Mor-stim Mic), ^†††^*P*=0.0003, 0.0001 (Mor-stim Mic versus Mor+Pax-stim Mic), (drug × time point interaction): F_3, 60_=20.77, a two-way ANOVA followed by the Bonferroni *post hoc* test (**a**). **P=0.0071, ***P=0.0001 (Saline versus Mor-stim Mic), ^††^*P*=0.0014, ^†††^*P*=0.0001 (Mor-stim Mic versus Mor+Pax-stim Mic), (drug × time point interaction): F_3, 60_=20.77, a two-way ANOVA followed by the Bonferroni *post hoc* test (**b**). ***P=0.0001, 0.0001 (*Slo1*^+/+^ Cont Mic versus *Slo1*^+/+^ Mor-stim Mic), ^††^*P*=0.0012, ^†††^*P*=0.0001 (*Slo1*^+/+^ Mor-stim Mic versus *Slo1*^−/−^ Mor-stim Mic), (drug × time point interaction): F_4, 75_=10.6, a two-way ANOVA followed by the Bonferroni *post hoc* test (**c**). **P=0.0023, 0.0079, ***P=0.0001 (*Slo1*^+/+^ Cont Mic versus *Slo1*^+/+^ Mor-stim Mic), ^††^*P*=0.0024, ^†††^*P*=0.0001, 0.0001 (*Slo1*^+/+^ Mor-stim Mic versus *Slo1*^−/−^ Mor-stim Mic), (drug × time point interaction): F_4, 175_=27.16, a two-way ANOVA followed by the Bonferroni *post hoc* test (**d**).

To further assess the significance of microglial BK channels in the generation of MIH and anti-nociceptive tolerance, we conducted adoptive transfer experiments using primary microglia^20^,^27^,^28^,^29^,^30^ that were exposed to 10 μM morphine for 24 h. Recipient mice subjected to i.t. transfer of morphine-stimulated primary microglia showed a significant facilitation of the generation of MIH and anti-nociceptive tolerance, whereas the i.t. transfer of non-stimulated primary microglia did not lead to any alterations (Fig. 2a,b and Supplementary Fig. 6a,b). The facilitated generation of MIH and anti-nociceptive tolerance was completely abrogated in the mice transferred primary microglia that had been stimulated with morphine in the presence of paxilline (2 μM) (Fig. 2a,b and Supplementary Fig. 6a,b). These phenomena were also observed when the mice were subjected to the i.t. transfer of morphine-stimulated *Slo1*-lacking primary microglia (Fig. 2c,d and Supplementary Fig. 6c,d). These results strongly suggest that microglial BK channels have a crucial role in the generation of MIH and tolerance. No further behavioural studies using *Slo1*^*−/−*^ mice were performed in the present study, because the *Slo1*^*−/−*^ mice were viable, but exhibited moderate ataxia^31^.

### AA pathway activates BK channels in microglia

**Figure 3:**
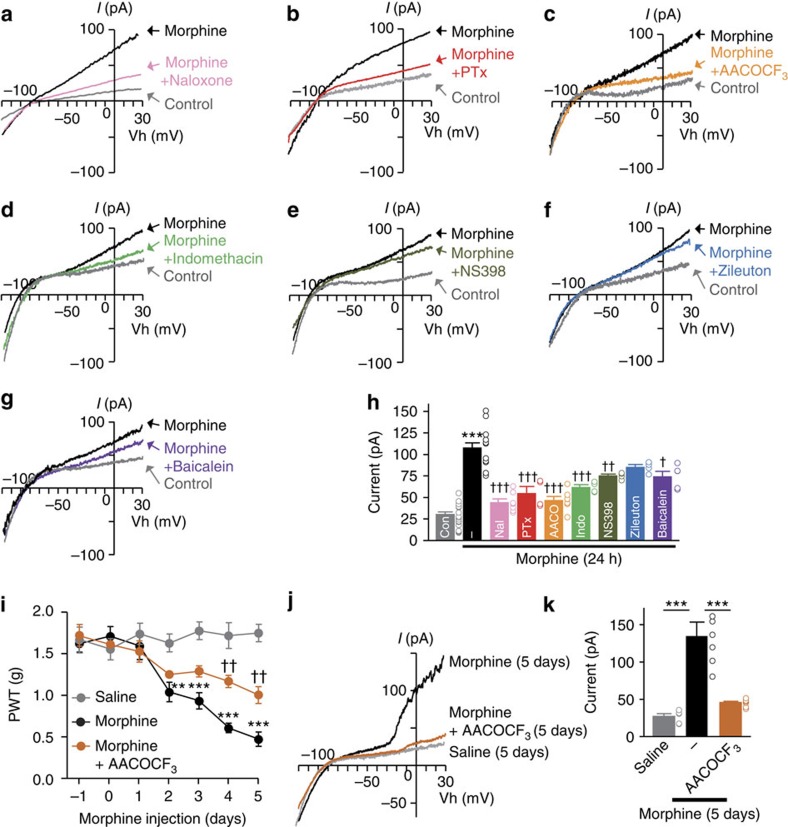
The PLA_2_–AA pathway triggers the current activation of BK channels in microglia following morphine treatment. (**a**–**g**) Typical traces of BK currents in MG6 after 24 h of morphine (10 μM) treatment with or without naloxone (a μ-opioid receptor inhibitor, 10 μM) (**a**), pertussis toxin (PTx, 10 nM) (**b**), AACOCF_3_ (10 μM) (**c**), indomethacin (1 μM) (**d**), NS398 (10 μM) (**e**), zileuton (100 μM) (**f**) and baicalein (100 μM) (**g**). (**h**) The amplitude of BK currents at +30 mV in MG6 after 24 h of morphine treatment (*n*=22/16/7/4/7/4/4/4/5 cells). (**i**) The PWT of morphine-treated mice with or without i.t. injection of AACOCF_3_ (100 pmol) (*n*=5 for Saline, *n*=5 for Morphine, *n*=6 for Morphine+AACOCF_3_). (**j**) BK currents in the lamina I spinal microglia after 5 days of morphine administration. (**k**) The amplitude of BK currents at +30 mV in the lamina I spinal microglia (*n*=5 for Saline, *n*=6 for Morphine, *n*=8 for Morphine+AACOCF_3_, from three mice each). The data represent the means±s.e.m. ***P=0.0001 (Cont versus Morphine), ^†^*P*=0.0101, ^††^*P*=0.0027, ^†††^*P*=0.0001, 0.0001, 0.0001, 0.0001 (versus Morphine), F_8, 64_=33.34, a one-way ANOVA followed by the Tukey's *post hoc* test (**h**), **P=0.0021, ***P=0.0001, 0.0001, 0.0001 (Saline versus Morphine), ^††^*P*=0.0027, 0.0084 (Morphine versus Morphine+AACOCF_3_), (drug × time point interaction): F_2, 16_=33.34, a two-way ANOVA followed by the Bonferroni *post hoc* test (**i**). ***P=0.0001, 0.0001 (each column), F_2, 91_=52.51, a one-way ANOVA followed by the Tukey's *post hoc* test (**k**).

We further explored the mechanism underlying morphine-induced BK channel activation in microglia by pharmacological methods. Morphine (10 μM, 24 h) elicited BK currents in MG6 microglial cell line (MG6), which are sensitive to bath application of BK channel inhibitors (Supplementary Fig. 1c–g), in a time-dependent manner (Supplementary Fig. 7a,b). The activation of BK channels at 24 h after morphine stimulation was significantly inhibited by naloxone (a μ-opioid receptor inhibitor) or pertussis toxin (a Gi protein inhibitor) (Fig. 3a,b). The μ-opioid receptors are known to be functionally coupled with phospholipase A_2_ (PLA_2_)^32^. As expected, cytosolic PLA_2_ (cPLA_2_) in MG6 was time-dependently phosphorylated after morphine stimulation (Supplementary Fig. 7c). Additional experiments showed that BK currents in MG6 elicited by morphine exposure for 24 h were inhibited by AACOCF_3_ (a PLA_2_ inhibitor) (Fig. 3c), suggesting the possible involvement of the AA pathway in the activation of BK channels in microglia. Extracellularly applied AA could evoke BK currents in MG6 in a dose-dependent manner (Supplementary Fig. 7d,e). Of note, current activation was elicited 4–5 min after AA (10 μM) application (Supplementary Fig. 7f). In contrast, the intracellular application of AA (10 μM) through the patch pipette immediately activated BK currents in MG6 (Supplementary Fig. 7g–i). The differential kinetics of BK current activation by AA suggests that AA activates the intracellular domain of BK channels in microglia.

In further pharmacological studies on BK channels in microglia, indomethacin (a COX1/2 inhibitor), NS398 (a COX2 inhibitor) and baicalein (a 12/15-lipoxygenase inhibitor) all significantly inhibited the BK channel activation in MG6 after morphine stimulation, while zileuton (a 5-lipoxygenase inhibitor) had only a weak effect on them (Fig. 3d–h). Unlike AA treatment, the direct application of prostaglandin E_2_ (PGE_2_) also activated BK channels in MG6, but only after intracellular perfusion (Supplementary Fig. 7j–n). The behavioural analyses showed that the i.t. injection of AACOCF_3_ significantly attenuated the generation of MIH and anti-nociceptive tolerance (Fig. 3i and Supplementary Fig. 8). Furthermore, on day 5 of morphine administration, BK currents in the lamina I microglia were significantly suppressed by the i.t. injection of AACOCF_3_ (Fig. 3j,k).

### Morphine increases synaptic transmission

**Figure 4:**
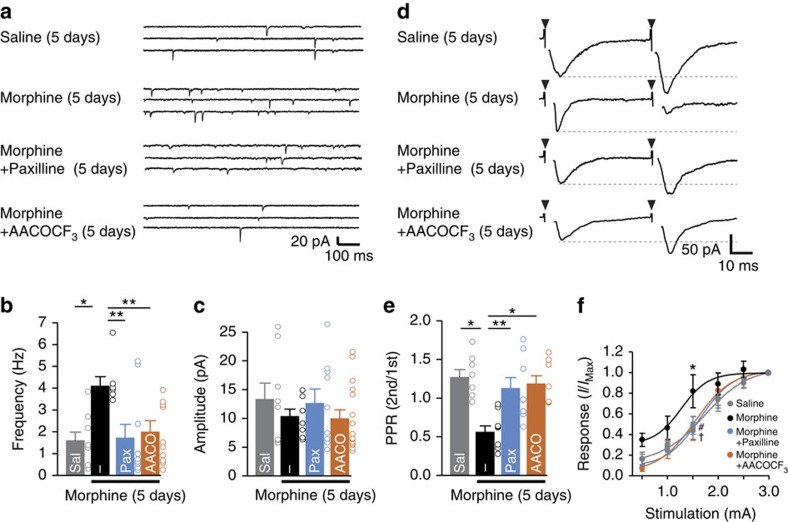
The recovery effects of BK channel or PLA_2_ inhibitors on the increased neurotransmission in lamina I neurons. (**a**) Typical traces of mEPSCs recorded from the lamina I spinal neurons after 5 days of saline or morphine (10 mg kg^−1^) treatment. BK channel (2.2 μg kg^−1^, i.p.) or PLA_2_ inhibitors (100 pmol, i.t.) were administered 30 min prior to morphine treatment. (**b**,**c**) The averaged frequency (**b**) and amplitude (**c**) of mEPSCs (*n*=8 from three mice for Saline, *n*=6 from three mice for Morphine, *n*=9 from three mice for Morphine+Paxilline, *n*=13 from four mice for Morphine+AACOCF_3_). Sal, Pax and AACO indicate saline, paxilline and AACOCF_3_, respectively. (**d**) Typical traces of dorsal root entry stimulated currents recorded from the lamina I spinal neurons after 5 days of morphine treatment. The triangles indicate dorsal root stimulation. A second stimulus was delivered 50 ms after the first. (**e**) The PPR was calculated as the ratio of the 2nd/1st responses. (**f**) The current–intensity curves of the dorsal root-evoked currents. The half values of *I*/*I*_MAX_ were 1.739 mA (Saline), 1.180 mA (Morphine), 1.652 mA (Morphine+Paxilline) and 1.571 mA (Morphine+AACOCF_3_). (*n*=7 for Saline, *n*=8 for Morphine, *n*=9 for Morphine+Paxilline, *n*=7 for Morphine+AACOCF_3_, from three mice each). The data represent the means±s.e.m. *P=0.0124 (Saline versus Morphine), **P=0.0062 (Morphine versus Morphine+Paxilline), **P=0.0091 (Morphine versus Morphine+AACOCF_3_), F_3, 31_=5.127, a one-way ANOVA followed by the Tukey's *post hoc* test (**b**). *P=0.0112 (Saline versus Morphine), **P=0.0035 (Morphine versus Morphine+Paxilline), *P=0.00110 (Morphine versus Morphine+AACOCF_3_), F_3, 31_=0.2830, a one-way ANOVA followed by the Tukey's *post hoc* test (**e**), *P=0.0445 (Saline versus Morphine), ^†^*P*=0.0427 (Morphine versus Morphine+Paxilline) and ^#^*P*=0.0404 (Morphine versus Morphine+AACOCF_3_), (drug × time point interaction): F_3, 162_=5.51, a two-way ANOVA followed by the Bonferroni *post hoc* test (**f**). Scale bars, 20 pA and 100 ms (**a**); and 50 pA and 10 ms (**d**).

Lamina I neurons respond to painful stimuli and are one of the main nociceptive pathways from the spinal cord to the brain^33^. Chronic morphine treatment can alter the neurotransmission of primary afferent terminals to the lamina I neurons^34^. We therefore analysed the associations between functional alterations in synaptic transmission and the attenuation of MIH. Patch clamp recordings were obtained from the lamina I neurons in the spinal cord slices prepared from mice after a 5-day treatment with morphine (10 mg kg^−1^). We observed as significant increase in the mean frequency, but not the mean amplitude, of the miniature excitatory postsynaptic currents (mEPSC) in the lamina I neurons after a 5-day treatment with morphine (4.1±0.43 Hz) compared with the saline-treated control (1.6±0.39 Hz) (Fig. 4a–c), similar to previous observations^34^. In contrast, repetitive administration of both paxilline and AACOCF_3_ could significantly suppress the increase in the mean frequency of mEPSC in the lamina I neurons after a 5-day treatment with morphine (Paxilline: 1.7±0.63 Hz, AACOCF_3_: 2.0±0.52 Hz) (Fig. 4a,b).

To further characterize the increased release probability of primary afferent to lamina I neurons, we recorded the currents evoked by the stimulation of dorsal root entry. The repeated responses from lamina I neurons evoked by stimulations at 50-ms intervals were calculated as the paired pulse ratio (PPR). At 5 days of morphine administration, the PPR was 0.56±0.08. In contrast, the PPR in the saline-treated group was 1.27±0.10 (Fig. 4d,e), which was significantly facilitated as compared with the morphine-treated group. The PPR recorded from spinal cord slices that were made when we observed the attenuation of MIH and anti-nociceptive tolerance with repetitive administration of paxilline or AACOCF_3_ significantly recovered to a level similar to the saline-treated control (Paxilline: 1.25±0.21, AACOCF_3_: 1.18±0.10). In addition, the current–intensity curve showed a leftward shift in the morphine-treated group compared with the saline-treated control (Fig. 4f). The curve-fitting analyses showed a significant difference in the half value of the *I*/*I*_MAX_. These observations suggest that microglia facilitate neurotransmission in primary afferent to the lamina I neurons following chronic morphine administration.

### The interrelationship between BK channels and P2X4Rs

**Figure 5:**
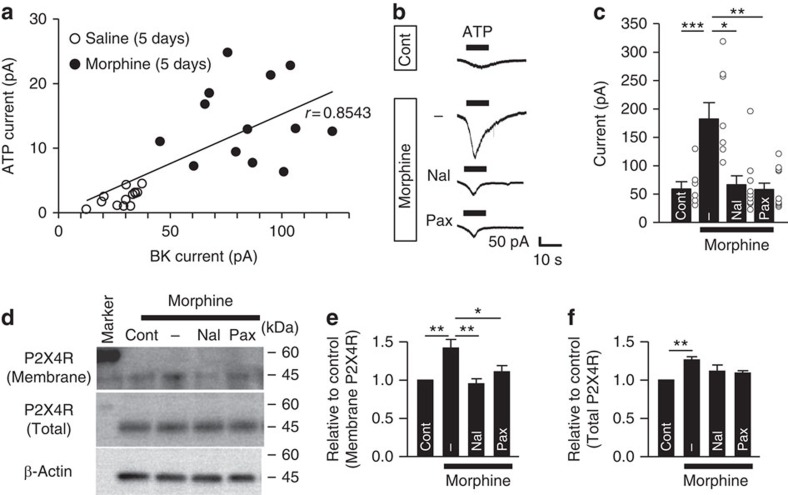
The BK channel-dependent membrane translocation of the P2X4Rs after morphine stimulation in microglia. (**a**) A scatter plot of both the ATP (3 mM)-induced currents and BK currents at +30 mV in the lamina I spinal microglia after 5 days of saline or morphine (10 mg kg^−1^) administration (two-tailed Spearman's test, *r*=0.8543, *P*<0.0001, *n*=13 for Saline, *n*=13 for Morphine, from four mice each). (**b**) Typical traces of ATP (3 mM)-induced currents in the MG6 after 24 h of morphine (10 μM) stimulation. Naloxone (Nal, 10 μM) or paxilline (Pax, 2 μM) was added 1 h prior to morphine treatment. (**c**) The peak amplitude of the ATP-induced inward currents at a holding potential of −60 mV (*n*=7/8/10/10 cells). (**d**) An immunoblot to examine the membrane translocation of P2X4Rs following stimulation with morphine (10 μM) for 24 h in primary microglia. The cells were exposed to morphine in the presence or absence of naloxone (Nal, 10 μM) or paxilline (Pax, 2 μM). (**e**,**f**) The P2X4R levels in the membrane fraction (**e**) or total lysates (**f**) were quantified relative to the control. The data represent the means±s.e.m. ***P=0.0005 (Control versus Morphine), 0.0003 (Morphine versus Morphine+Naloxone), 0.0001 (Morphine versus Morphine+Paxilline), F_3, 31_=10.87, a one-way ANOVA followed by the Tukey's *post hoc* test (**c**). *P=0.0281 (Control versus Morphine), **P=0.0032 (Control versus Morphine), 0.0010 (Morphine versus Morphine+Naloxone), F_3, 24_=7.824, a one-way ANOVA followed by the Tukey's *post hoc* test (**e**). **P=0.0057 (Control versus Morphine), F_3, 24_=4.956, a one-way ANOVA followed by the Tukey's *post hoc* test (**f**). Scale bar, 50 pA and 100 ms (**b**).

Since microglial P2X4Rs have a pivotal role in the pathogenesis of MIH and anti-nociceptive tolerance^19^,^20^, BK channels might have a functional relationship with P2X4Rs in the spinal microglia. Following the recording of BK currents, ATP-induced currents were recorded from the same microglia in the lamina I spinal cord. The resulting scatter plot exhibited a strong positive correlation between the ATP-induced inward currents and BK currents after morphine administration (*r*=0.8543, Spearman's test, Fig. 5a). Further analyses of the positive interaction between ATP-induced currents and BK currents were conducted using MG6. The ATP-induced inward currents were significantly enhanced by the stimulation with morphine (10 μM) for 24 h (Fig. 5b,c), and were sensitive to *O*-(2,4,6-trinitrophenyl) adenosine 5-triphosphate (TNP-ATP), an antagonist of P2XR subtype P2X1–4R. In contrast, pyridoxal-phosphate-6-azophenyl-2′,4′-disulphonic acid (PPADS), an antagonist of P2XR subtypes P2X1, 2, 3, 5, 7R, but not of P2X4R, had no effect on the ATP-induced currents (Supplementary Fig. 9), suggesting that morphine enhanced the P2X4Rs-mediated currents. This event was significantly suppressed by naloxone (Fig. 5b,c). It is noteworthy that paxilline suppressed the morphine-induced increase in P2X4R currents (Fig. 5b,c), suggesting that BK channels regulate the membrane trafficking of P2X4Rs from the lysosomal membrane^35^.

To address this possibility, membrane proteins in primary microglia were collected by biotinylation, and the specimens were subjected to immunoblot analyses. Morphine stimulation significantly increased the expression of both the membrane and the total protein of P2X4Rs (Fig. 5d–f). Both naloxone and paxilline could inhibit the increase in the mean membrane protein level of P2X4Rs in primary cultured microglia stimulated by morphine (Fig. 5d–f). The *Slo1*-ablation significantly suppressed the morphine-induced increased expression of the membrane P2X4Rs in primary microglia (Supplementary Fig. 10b). These observations clearly indicate that the activation of BK currents facilitates the membrane trafficking of P2X4Rs in microglia.

### Possible link between BK channels and secretory pathways

**Figure 6:**
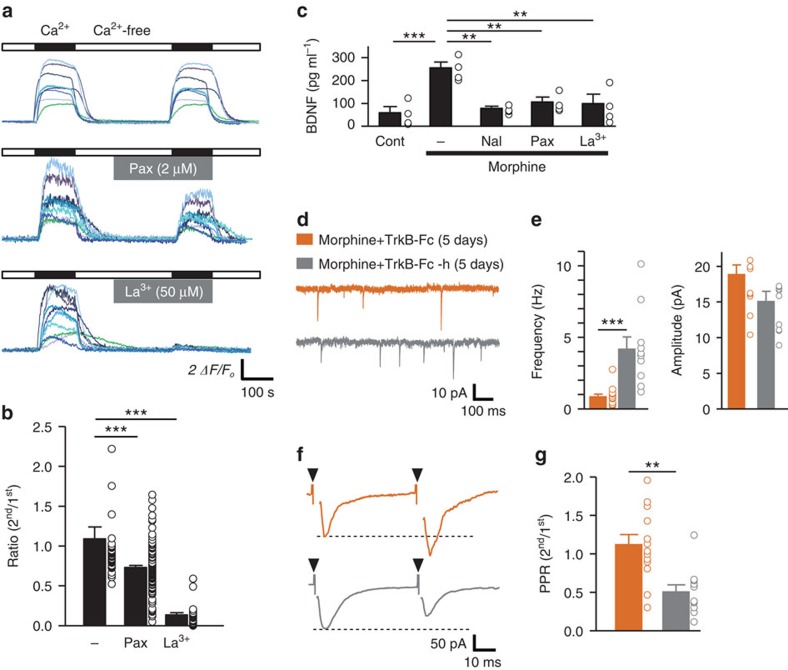
BK channels provoke an ionic imbalance through the activation of store-operated Ca^2+^ entry. (**a**) Ten superimposed traces of the calcium fluorescence response of MG6 after 24 h of morphine (10 μM) stimulation. Closed and open bars indicate the application of 1 mM Ca^2+^ solution and Ca^2+^-free solution, respectively. Grey bars indicate the application of paxilline or LaCl_3_, respectively. (**b**) The ratio of the 2nd/1st peak Δ*F/F*_*0*_ responses (*n*=50/122/33 cells). (**c**) The measurement of the BDNF in the supernatant of MG6 following stimulation with morphine (10 μM) for 24 h. Naloxone (Nal, 10 μM), paxilline (Pax, 2 μM) or LaCl_3_ (La^3+^, 50 μM) was added 1 h prior to morphine treatment (*n*=4 independent experiments). (**d**,**f**) Typical traces of the mEPSCs and root-evoked EPSCs recorded from the lamina I spinal neurons after 5 days of morphine administration in mice. TrkB-Fc (10 ng) and TrkB-Fc-h (heat-inactivated TrkB-Fc,10 ng) were intrathecally injected 1 h prior to morphine treatment. (**e**) The averaged frequency and amplitude of the mEPSCs (*n*=12 for TrkB-Fc, *n*=10 for TrkB-Fc-h, from three mice each). (**g**) The ratio of 2nd/1st peak currents (*n*=13 for TrkB-Fc, *n*=11 for TrkB-Fc-h, from three mice each). Orange and grey traces and columns indicate Morphine+TrkB-Fc (5d) and Morphine+TrkB-Fc-h (5d), respectively (**d**–**g**). The data represent the means±s.e.m. ***P=0.0001, 0.0001 (each column), F_2, 201_=94.3, a one-way ANOVA followed by the Tukey's *post hoc* test (**b**). **P=0.0016, 0.0073, 0.0035 (each column), ***P=0.0006, F_4, 15_=9.351, a one-way ANOVA followed by the Tukey's *post hoc* test (**c**). ***P=0.0005, *t*(20)=4.169 (**e**, left), *P*=0.0715, *t*(20)=1.904 (**e**, right), an unpaired *t*-test (**e**). **P=0.0013, *t*(22)=3.684, an unpaired *t*-test (**g**).

Based on the above findings, it is reasonable to consider that BK channels indirectly modify the intracellular Ca^2+^ concentration ([Ca^2+^]_*i*_), because the [Ca^2+^]_*i*_ is responsible for membrane trafficking. It was previously suggested that the Ca^2+^ influx following the activation of BK channels is associated with SOCE^36^. Therefore, we performed intracellular Ca^2+^ imaging. MG6, which was exposed to morphine for 24 h, was loaded with Fluo-4-AM, a calcium indicator dye. To directly observe SOCE, thapsigargin and nifedipine were included in the external solution. An extracellular perfusion of 1 mM Ca^2+^ solution evoked a rapid rise in the [Ca^2+^]_*i*_ in MG6, which was completely blocked by La^3+^, an inhibitor of SOCE (Fig. 6a,b). This robust Ca^2+^ influx after morphine stimulation for 24 h was significantly inhibited by bath application of paxilline in MG6 and primary microglia (Fig. 6a,b and Supplementary Fig. 11a–c). In addition, *Slo1* gene-silenced MG6 and primary microglia exhibited a weak Ca^2+^ influx which was less sensitive to paxilline after morphine stimulation (Supplementary Fig. 11d–f). Conversely, the bath application of AA dramatically potentiated Ca^2+^ influx in non-stimulated MG6, which was not observed after morphine stimulation (Supplementary Fig. 11g–k), implying that the intracellular AA within microglia had already been saturated after morphine stimulation. These results suggest that the K^+^ efflux through BK channels after stimulation with morphine conversely promotes the Ca^2+^ influx.

We further analysed the linkage between BK channels and the secretory pathway by focusing on the P2X4Rs–BDNF axis^20^,^28^,^37^. The morphine-induced BDNF secretion from MG6 was significantly suppressed by naloxone, paxilline and La^3+^ (Fig. 6c). These results indicated that the BK channel-mediated imbalance of electrical homeostasis facilitates the secretion of BDNF from microglia. We next explored the importance of BDNF on the synaptic transmission in the lamina I neurons. Morphine-treated mice were subjected to TrkB-Fc (10 ng, i.t.), a chimeric binding protein which sequesters BDNF, and subsequently whole-cell recording from the lamina I neurons were performed. TrkB-Fc led to a mEPSC frequency of 0.86±0.20 Hz and a PPR of 1.11±0.13 (Fig. 6d–g), which were similar to the values in saline-treated mice (Fig. 4). In contrast, heat-treated TrkB-Fc (10 ng, i.t.) did not lead to any significant difference in the mEPSC frequency (4.28±0.87 Hz) and PPR (0.50±0.09) compared with the morphine-treated mice (Figs 4 and 6d–g).

### KCNMB3 of BK channel subunit in microglia involves MIH

**Figure 7:**
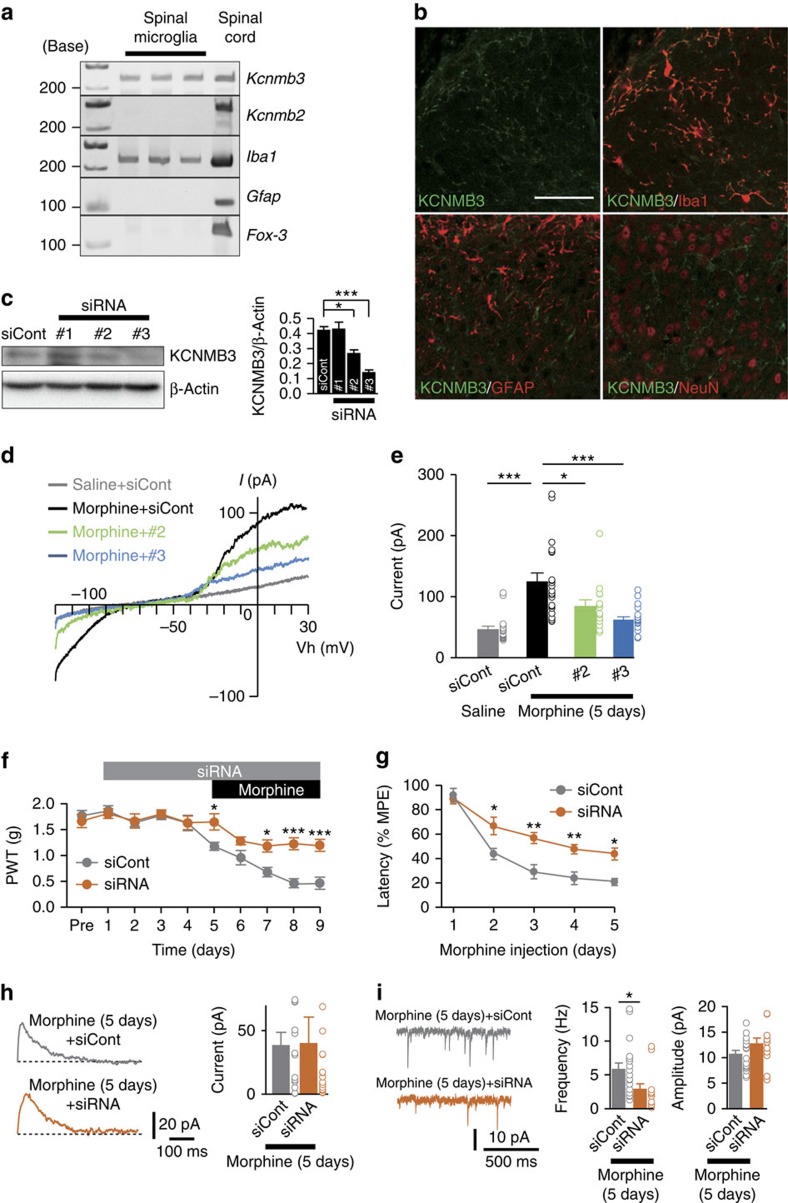
Gene silencing of Kcnmb3 abrogates MIH and anti-nociceptive tolerance. (**a**) The northern blot of Kcnmb3 in single microglia collected from the lamina I spinal cord. (**b**) The immunofluorescence of KCNMB3 in the spinal dorsal horn. The upper panels show KCNMB3 (green) immunofluorescence and merged images with Iba1 immunofluorescence (red). The lower panels show merged images of KCNMB3 and GFAP or NeuN Alexa Fluor 555. (**c**) The immunoblot of KCNMB3 in the L4 dorsal spinal cord after 4 days of i.t. injection of KCNMB3 siRNA. The columns represent the relative expression of KCNMB3. (**d**) Typical traces of BK currents in the lamina I spinal microglia recorded after 5 days of morphine treatment. siCont or #2 or #3 siRNA was intrathecally injected 4 days before and during morphine treatment. (**e**) A comparison of the BK current amplitudes in the lamina I spinal microglia (*n*=18 for Saline+siCont, *n*=21 for Morphine+siCont, *n*=14 for Morphine+#2, *n*=21 for Morphine+#3). (**f**,**g**) The attenuation of hyperalgesia (**f**) and tolerance (**g**) following the i.t. injection of #3 KCNMB3 siRNA (*n*=5 for siCont and *n*=6 for siRNA). (**h**,**i**) BK currents (**h**) and mEPSCs (**i**) recorded from the lamina I spinal neurons after 5 days of morphine administration. siCont or siRNA was intrathecally injected 4 days before and during morphine administration (*n*=14 for siCont and *n*=14 for siRNA (**i**), *n*=17 for siCont and *n*=12 for siRNA (**j**), from three mice each). The data represent the means±s.e.m. *P*=0.0281, 0.0008 (each column), F_3, 8_=21.06, a one-way ANOVA followed by the Tukey's *post hoc* test (**c**). *P=0.0158, 0.0001, 0.0001 (each column), F_3, 70_=15.12, a one-way ANOVA followed by the Tukey's *post hoc* test (**e**). *P=0.0311, 0.0158, ***P=0.0001, 0.0001 (siCont versus siRNA), F_1, 90_=28.44, a two-way ANOVA followed by the Bonferroni *post hoc* test (**f**). *P=0.0119, ***P=0.0017, **P=0.0082, 0.0092 (siCont versus siRNA), F_1, 45_=35.12, a two-way ANOVA followed by the Bonferroni *post hoc* test (**g**). *P*=0.1920, *t*(26)=1.339, an unpaired *t*-test (**h**). *P=0.0357, *t*(26)=1.339, *P*=0.1342, *t*(27)=2.21 (**i**, left), *t*(27)=1.544 (**i**, right) an unpaired *t*-test (**i**). Scale bar, 50 μm (**b**).

To address the mechanism underlying the specific activation of BK channels in microglia, we focused on the AA-responsive properties of BK channels. BK channels are formed by the pore-forming α subunit (*Slo1*) and auxiliary β subunits (β1–β4), which comprise a heterotetramer. The AA-responsive patterns^38^ lead us to deduce that β2- or β3-containing BK channels might be expressed in the lamina I microglia. We therefore extracted GFP-positive microglia from the lamina I spinal cord with a patch pipette, and then performed single-cell PCR analyses. All GFP-labelled cells were Iba1 positive, which never expressed mRNA for GFAP, an astrocyte-specific gene, or Fox-3, a neuron-specific gene^39^ (Fig. 7a). As expected, *Kcnmb3* (β3 subunit) mRNA was expressed in microglia not in astrocytes and neurons in the lamina I spinal cord (Supplementary Fig. 12c). Other subtypes of *Kcnmb* mRNA were not expressed in the lamina I microglia, MG6 and primary microglia (Supplementary Fig. 12). Of note, KCNMB3 was exclusively expressed in the spinal microglia (Fig. 7b), however, a weak KCNMB3 signal was observed in the blood vessel-like structures in the deep laminae of the spinal cord (Fig. 7b and Supplementary Fig. 13). These observations indicate that KCNMB3 is a microglia-specific subtype in the superficial laminae of the spinal cord.

To further test whether microglia-specific KCNMB3 is responsible for the generation of MIH and anti-nociceptive tolerance, we conducted *in vivo* gene silencing of KCNMB3, the microglia-specific subtype, using a small interfering RNA (siRNA). We used three kinds of siRNA for KCNMB3, which were delivered by the i.t. injection once daily for 4 days to achieve the selective depletion of KCNMB3 in the spinal dorsal horn of naive mice. The KCNMB3 protein was expressed in the L4 spinal dorsal horn of naive mice, although the mean level of it was low (Fig. 7c). The amount of KCNMB3 protein in the L4 of the dorsal spinal cord was not affected by the chronic morphine administration (Supplementary Fig. 14). Likewise, the *Kcnmb3* mRNA was expressed at a much lower level in the spinal cord than in the testes (a positive control) (Supplementary Fig. 15). Both the #2 and #3 KCNMB3 siRNA (20 pmol, i.t.) could deplete the KCNMB3 protein from the L4 spinal dorsal horn, whereas the #1 siRNA could not (Fig. 7c). Based on the results of the immunoblot analyses, we utilized the #2 and #3 siRNA in the subsequent patch clamp analyses of the lamina I microglia. The gene silencing of KCNMB3 with the #2 and #3 siRNA significantly inhibited the morphine-induced BK current activation (Fig. 7d,e). In particular, a remarkable inhibition of BK currents was observed by the #3 siRNA (i.t.), which caused the significant depletion of *Kcnmb3* mRNA (Supplementary Fig. 15). The channel property of KCNMB3, which was activated at voltages below 0 mV at a given Ca^2+^ concentration^40^ was abolished by #3 siRNA (i.t.) in the lamina I microglia (Supplementary Fig. 16).

Finally, we investigated the effects of KCNMB3 silencing by the #3 siRNA on pain behaviour. KCNMB3 ablation markedly attenuated the generation of MIH and anti-nociceptive tolerance (Fig. 7f,g). However, KCNMB3 siRNA itself did not affect the basal nociception. Gene silencing of KCNMB3 had no obvious effect on the neuronal BK currents (Fig. 7h). More interestingly, KCNMB3 ablation in the lamina I microglia markedly inhibited the morphine-elicited increase in neurotransmission (Fig. 7i), indicating that specific activation of BK channels in microglia by chronic morphine treatment is responsible for abnormal neurotransmission in the spinal cord.

## Discussion

BK channels are usually distributed throughout the central nervous system (CNS) and regulate the neuronal excitability. Therefore, the activation of BK channels is generally believed to serve as an ‘emergency brake' that would regulate excessive generation of action potentials or transmitter release^41^. Indeed, the lamina I neurons of *Slo1*^*−/−*^ mice exhibited excessive neurotransmission (Supplementary Fig. 17) in comparison to WT mice (Fig. 4). Interestingly, the function of BK channels in microglia seems to be strikingly different from that in neurons. Microglia are electrically non-excitable cells, because they never generate action potentials due to their lack of sodium channels^42^. We previously reported that the activation of BK channels in spinal microglia causes the tactile allodynia^18^. However, there is a discrepancy in the role of BK channels in the sensation of pain. In contrast to our study, Chen *et al*.^43^, reported that the administration of a BK channel activator reduced nerve injury-induced allodynia in a dose-dependent manner. Conversely, in our study, an ultra-low dose of NS1619 (0.072 μg, i.t.) induced tactile allodynia and activated the spinal microglia in naive mice^18^. Similar to Chen's report, NS1619 (20 μg, i.t.) had no effects on the pain behaviour of naive mice^18^. The concentration of the BK channel activator may have been involved in this discrepancy^18^. However, the mechanism underlying the contribution of microglial BK channels to cellular activation was unclear. In the present study, we found an unique function for microglial BK channels. Robust K^+^ efflux as a consequence of the activation of BK channels following morphine stimulation conversely facilitated the driving force for Ca^2+^ entry through the SOCE, similar to a previous observation^36^. This Ca^2+^ influx was found to trigger the membrane translocation of P2X4Rs and BDNF synthesis. The functional membrane-inserted P2X4Rs play a key role in the synthesis of BDNF in the lamina I microglia, resulting in the enhancement of perceived pain^20^,^28^. Interestingly, the interrupting of the p38 MAPK pathway, which is downstream signalling of the P2X4Rs signal, partially suppressed the morphine-induced BK channel activation in the lamina I microglia (Supplementary Fig. 18). In contrast, they were not affected by the TLR4-mediated pathway (Supplementary Fig. 18), because of the weak relationship between TLR4 and MIH^20^,^23^,^44^. These results suggest that the BK channel-mediated cascade further activates microglia through BK channels. The partial amelioration of established MIH by paxilline (Supplementary Fig. 19) might break the vicious cycle of the P2X4Rs–BDNF axis in microglia^20^,^28^,^37^.

BK channels are generally activated by [Ca^2+^]_*i*_ or membrane demoralization^41^. In the present study, the BK channel activation preceded the activation of P2X4Rs or SOCE, which is a well-known Ca^2+^-permeable apparatus on the membrane surface. Therefore, it is an important issue to clarify how microglial BK channels are activated following morphine treatment. It has been reported that BK channels are basically activated by a >1 μM [Ca^2+^]_*i*_ (ref. 41). In the present study, we recorded BK currents from the lamina I microglia with a Ca^2+^-free pipette solution during the whole-cell configuration. Theoretically, BK currents are never activated under this condition. However, AA could activate BK channels even at extremely low [Ca^2+^]_*i*_ (0.1 μM)^45^. In fact, a cPLA_2_ inhibitor markedly suppressed the morphine-induced current activation of BK channels in the spinal microglia and MG6. Even under physiological conditions, a robust activation of BK currents was observed with a 1 μM of internal Ca^2+^ concentration compared with a 0.1 μM concentration in the lamina I microglia (Supplementary Fig. 20). This indicates that BK channels are functionally expressed even in normal microglia. Thus, AA drives the activation of BK channels even at low concentrations of [Ca^2+^]_*i*_.

AA may also be produced by neurons. However, it never activates the neuronal BK channels due to the mechanism discussed below. The activation of the CGRP, NK-1 and NMDARs triggers the synthesis of AA metabolites such as prostaglandins and lipoxygenase metabolites^46^. These factors are liberated and have a retrograde influence on presynaptic activity^47^,^48^. Consequently, the quantal size of the neurotransmitters from the primary afferent is facilitated. On the contrary, the intracellular perfusion of PGE_2_ and AA metabolites elicited BK currents in MG6, whereas extracellular perfusion did not (Supplementary Fig. 7j–n). This indicates that the microglia-derived AA metabolites activated the microglial BK channels. Distinct AA pathways yield individual bioactivity. The AA synthesis is mediated by the activation of opioid receptors, which stimulates MAPK activity^49^ and, in turn, activates PLA_2_ (refs 32, 50). AA metabolites produced by a COX-mediated pathway are responsible for the activation of BK channels in microglia. In the present study, the morphine-induced BK channel activation in microglia was significantly inhibited by COX inhibitors, but only partially inhibited by LOX inhibitors. Even after the shunting of COX-mediated pathway by COX inhibitors, AA can still be metabolized by the alternative LOX-mediated pathway^51^. Furthermore, a distinct potency of COX inhibitors and PLA_2_ inhibitor may suggest that AA and PGE_2_ additively or synergistically activate BK channels in microglia after chronic morphine stimulation. On the basis of these observations, AA derived from microglia is mainly responsible for activation of BK channels in microglia. Consequently, the production of AA after morphine treatment serves as an instigator of microglial activation through BK channel activation.

The question remains regarding how microglial BK channels can be specifically activated by chronic morphine treatment. Unlike the ubiquitous distribution of the α subunit (Slo1 or KCNMA1), tissue-specific expression patterns of β subunits determine the BK channel diversity, such as the Ca^2+^ sensitivity or channel kinetics^52^.We found that the lamina I astrocytes and neurons in the spinal cord expressed β2 and β4 subunits, respectively (Supplementary Fig. 12). These observations were partially consistent with previous findings^53^,^54^. Thus, neuronal BK channels in the lamina I spinal cord did not respond to AA, based on the channel properties of the BK channel auxiliary β subunits^38^. In contrast, microglia that express β subunits remain to be determined. In the present study, we found that microglia specifically express the β3 subunits. However, the amount of β3 subunits in the spinal cord is extremely low compared with that in the testes. Similarly, the expression levels of the β3 subunit in the human CNS were also low, however, this subunit is definitely expressed in the CNS^55^,^56^,^57^. Sun *et al*.^38^ suggested that expression of the β3 is less robust and probably more specific to certain cell types. A small population of microglia that comprise <10% of the total brain cells probably make it more difficult to detect the β3 subunit in the CNS.

One of the prominent features of the β3 subunits is their rapid inactivation. Movement of the β3 subunit N terminus into position enables an intimate association with the α subunit to occupy the BK channel pore^52^. The gating property of α/β3 BK channels in microglia may maintain the intracellular ionic homeostasis. Once microglia are activated by a stimulus, the microglial BK channels may become out of control due to the potentiation or loss of the inactivation of the β3 subunit. In the present study, we were unable to observe any alterations in the basal nociception by silencing of the β3 subunit. This indicates that the microglial activation state is not determined solely by the depletion of this subunit, and is instead due to the deficiency of a stimulus under physiological conditions. BK channels are an effective therapeutic target in patients with MIH. However, morphine-induced excessive neurotransmission in lamina I spinal cord was not attenuated in *Slo1*^*−/−*^ mice (Supplementary Fig. 17). The specific inhibition of molecular targets such as β3 subunits in microglia might be a potential therapeutic approach for MIH. The generation of mice in which KCNMB3 production in microglia is knocked out will improve the understanding of MIH.

The sensitization of NMDARs in the spinal neurons is a well-established mechanism for the generation of MIH and anti-nociceptive tolerance^2^,^3^,^4^,^5^,^6^. Nevertheless, there is strong evidence for the involvement of other molecules in the generation of MIH and anti-nociceptive tolerance^19^,^20^,^21^. Microglia-mediated mechanisms are attributed to the modulation of neurotransmission via NMDARs. Several molecules from microglia^20^,^58^,^59^ permit the enhancement of NMDAR function. Cytokines are a prominent factor. We also found that the morphine-induced increased productivity of interleukin-1β in the spinal microglia was significantly suppressed by the supplementation with *S*-ketamine or a BK channel inhibitor (Supplementary Fig. 21). Thus, the inhibitory effect of microglial activation might be mediated by BK channels in spinal microglia.

**Figure 8:**
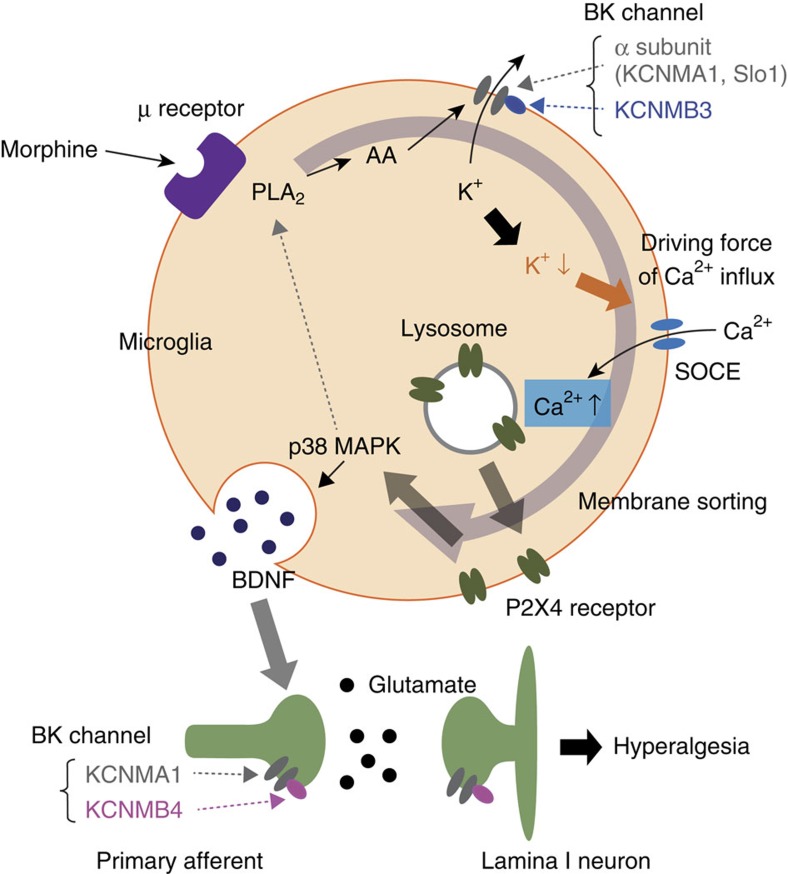
A schematic illustration of the proposed model. Morphine activates BK channels in microglia. The increase in the levels of intracellular arachidonic acid (AA) and its metabolite via the activation of PLA_2_ facilitate the gating of BK channels. The robust efflux of K^+^ results in reduction of the intracellular cation concentration, which triggers the driving force of the Ca^2+^ influx via store-operated Ca^2+^ entry (SOCE). An increase in intracellular Ca^2+^ facilitates membrane sorting of P2X4Rs from lysosomes. Consequently, the synthesis of BDNF by microglia potentiates neurotransmission in lamina I spinal neurons. BK channels in microglia therefore play a key role in the vicious cycle after morphine stimulation.

However, *S*-ketamine is a well-known NMDAR antagonist. Pharmacological analyses revealed that ∼1 μM of *S*-ketamine inhibited the NMDARs^18^. On the other hand, *S*-ketamine with the same concentration also could inhibit BK currents recorded from the lamina I microglia (Supplementary Fig. 20d) in a similar manner reported previously^60^, indicating that *S*-ketamine exhibits almost the same selectivity for the inhibition of NMDARs and BK channels. Therefore, the present study has led to a new idea that *S*-ketamine can ameliorate MIH through the inhibition of both NMDARs in the spinal neurons and BK channels in the spinal microglia. In the present study, we did not observe complete improvement of the MIH and anti-nociceptive tolerance with the loss-of-function of BK channels in microglia. Mechanism involving the desensitization of μ-opioid receptors^61^ or inhibition of glycine receptor subtype α3 by PGE_2_ (ref. 62) cannot be ruled. However, the serious side effects associated with the inhibition of NMDARs worsen the quality of life (QOL). Thus, supplementation with an ultra-low dose of *S*-ketamine or BK channel inhibitors might effectively suppress the amplification machinery of NMDARs by inhibiting microglial activation (Fig. 8) and would also decrease the occurrence of the serious adverse effects associated with opioid analgesics. The microglia-specific auxiliary β3 subunit thus becomes a promising therapeutic target for microglial channelopathies such as MIH and opioid anti-nociceptive tolerance.

## Methods

### Animals

Ten-week-old male C57BL/6 mice (Clea, Japan), *Iba1*^*+/GFP*^ mice^63^, and BK channel-deficient mice (*Slo1*^−/−^)^31^ and their wild-type (*Slo1*^*+/+*^) littermates were used for the experiments. Two- to three-day-old Wister rats (Kyudo, Japan) were used to obtain the primary cultured microglia. The mice were maintained on a 12-h light/dark cycle (light on 0800 hours) under conditions of 22–25 °C ambient temperature with food and water provided ad libitum. All mice were handled daily for 5 days before the start of the experiment to minimize their stress reactions to manipulation. The experimental protocol was approved by the Animal Research Committee of Kyushu University.

### Drug administration

All the drugs were purchased from Sigma, unless otherwise indicated. I.t. injection was performed using a 25 μl Hamilton syringe with a 30 gauge needle under isoflurane (2%) anaesthesia^18^. A reflexive flick of the tail was observed when the needle was inserted into the intervertebral space. Iberiotoxin (1 pmol), apamin (1 pmol), NS1619 (0.2 pmol), AACOCF_3_ (100 pmol), a recombinant human TrkB-Fc chimeric protein (10 ng, R&D Systems), heat-inactivated (100 °C for 10 min) recombinant human TrkB-Fc chimeric protein (TrkB-Fc-h, 10 ng) or sulforhodamine 101 (SR101; 1.5 μg, Life Technologies) were all injected i.t. 30 min prior to morphine administration. All the reagents dissolved in saline and 5 μl of the solution was injected i.t. KCNMB3 siRNA (Life Technologies, #1: MSS224455, #2: MSS224454, #3: MSS284619) or control siRNA (GC duplex negative control) was suspended in Lipofectamine RNAiMAX (Thermo Fisher Scientific). The KCNMB3 siRNA (20 pmol, 5 μl) was i.t. injected for 4 days prior to the experiment. Paxilline (2.2 or 4.4 μg kg^−1^), *S*- or *R*-ketamine (2 mg kg^−1^), ketamine racemate (2 mg kg^−1^) or minocycline (20 mg kg^−1^) were injected i.p. All of the drugs were injected 30 min before morphine administration. KCNMA1 (Slo1) siRNA (Thermo Fisher Scientific, MSS205723, 5 pmol) was applied to MG6 and primary microglia for 24 h.

### Behavioural analyses

All mice were habituated to the testing environment for 3 days. Morphine hydrochloride (10 mg kg^−1^; Daiichi Sankyo Propharma, Japan) was injected i.p. twice a day (1000 and 2100 hours) for 5 days into C57BL/6 mice and *Iba1*^*+/GFP*^ mice. The mechanical pain threshold was measured before morphine treatment using von Frey filaments (0.02–2.0 g; North Coast Medical) which were applied to the mid-plantar surface of the hindpaw^18^. The 50% paw withdrawal thresholds were calculated using the up–down method. The thermal pain threshold was measured before and after 1 h of morphine treatment using a hot plate apparatus (MK-350D, Muromachi Kikai Co., Tokyo, Japan). Each mouse was placed on a hot plate at 55 °C and the latency of the response to the heat stimulus was measured. The latency to paw licking or jumping was used as the dependent measure. A cutoff time of 30 s was chosen to minimize the thermal damage to mice. The reported data account for this artificial ceiling, as well as for the basal responsiveness of each mouse to the test is presented as the percentage of maximum possible effect (%MPE), which is calculated by the following formula: 100% × [(drug response time—basal response time)/(30 s−basal response time)]=%MPE. Rotarod testing was performed with an automated single-lane rotarod treadmill (Muromachi; 3-cm diameter drums with grooves to improve the grip) as reported previously^18^. The time when the mouse fell from the rod was recorded, with the maximum time being 300 s. All of the behaviours were tested blindly.

### Cell culture

Primary cultured microglia from C57BL/6 mice and *Slo1*^+/+^ and *Slo1*^−/−^ mice were prepared from neonatal cortex^18^,^59^. After removable of meninges and bloods, the tissue was minced with a razor blade. And the tissue was incubated with PBS containing bovine serum albumin (2 mg ml^−1^), DL-Cysteine Hydrochloride (2.23 mg ml^−1^), glucose (50 mg ml^−1^), papain (90 U ml^−1^, Worthington) and DNase (2,000 U ml^−1^, Worthington) at 37 °C for 15 min. To terminate the reaction, foetal bovine serum (FBS) was added. The tissue was suspended with Eagle's MEM medium (Nissui) containing 0.5% NaHCO_3_, penicillin–streptomycin (10,000 U ml^−1^, Gibco), L-glutamine (30 mg ml^−1^) and 10% FBS. After 10–14 days in culture, floating cells and weakly attached cells on the mixed glial cell layer were isolated by shaking the flask. The resultant cell suspension was then transferred to dish and allowed to adhere at 37 °C for 30 min. On the following day, primary microglia were subjected to morphine (10 μM) exposure with or without paxilline (2 μM) for 24 h. The cells were carefully washed with PBS and collected. A total of 1,000 cells per 10 μl were i.t. injected into naive mice^20^,^27^,^28^,^29^,^30^. Primary microglia from Wister rats were used to analyse the protein levels of P2X4R. A c-*myc*-immortalized mouse microglial cell line, MG6 (refs 64, 65; RIKEN BioResource Center: Cell No. is RCB2403), was maintained in a growth medium composed of DMEM containing 10% FBS (MP Biomedicals) supplemented with 100 μM β-mercaptoethanol, 10 μg ml^−1^ insulin, 100 μg ml^−1^ streptomycin and 100 U ml^−1^ penicillin in 100 mm Petri dishes^18^. MG6 was identified by anti-Iba1 rabbit polyclonal antibody, a marker for microglia. MG6 was subjected to 24-h exposure to morphine (10 μM) with or without naloxone (10 μM), pertussis toxin (10 μM), AACOCF_3_ (10 μM), indomethacin (1 μM), NS398 (10 μM), zileuton (100 μM) or baicalein (100 μM). No signs of mycoplasma contamination were observed by nuclear staining.

### Biotinylation of cell surface receptors

Primary cultured rat microglia were rinsed twice with ice-cold PBS and incubated with freshly prepared Sulfo-NHS-SS-biotin (Pierce) for 30 min at 4 °C. The cells were subsequently lysed in 500 μl of lysis buffer containing a protease inhibitor cocktail and were sonicated to further disrupt and homogenize the cells. After centrifugation of the resultant lysates, 50 μl of the supernatant was subjected to SDS–PAGE to analyse the total proteins. The biotinylated proteins in the remaining 450 μl were incubated with Immobilized NeutrAvidin Gel (Pierce) for 1 h at room temperature. After being washed, the proteins bound to the streptavidin beads were eluted in Laemmli buffer with DTT (20 μM) and subjected to SDS–PAGE, and were analysed for cell surface proteins.

### Western blot analysis

MG6 microglial cells or the L4-L5 spinal dorsal horn cells were collected after morphine or siRNA treatment. The specimens were lysed in lysis buffer (10 mM Tris-HCl (pH 7.4), 150 mM NaCl, 1% Triton X-100, 0.5% NP-40, phosphatase and protease inhibitor cocktail) and mixed with Laemmli sample buffer. Proteins (30 μg) were loaded into each lane and separated by 7.5% or 12% SDS–PAGE gel. After transfer, the blots were incubated overnight at 4 °C with an anti-cPLA_2_ rabbit polyclonal antibody (1:1,000; Cell Signaling, #2832), anti-phospho cPLA_2_ rabbit polyclonal antibody (1:1,000; Cell Signaling, #2831), anti-P2X4R rabbit polyclonal antibody (1:2,000; Alomone, APR-002), anti-KCNMB3 mouse monoclonal antibody (1:2,000; NOVUS, NBP2–12916) or anti-β-actin mouse monoclonal antibody (1:5,000; Abcam, ab8226). The primary antibodies were diluted in Can Get Signal Solution 1 (Toyobo). After being washed, the membranes were incubated with horseradish peroxidase-conjugated secondary antibody (1:1,000; GE Healthcare) for 1 h at room temperature. The membrane-bound horseradish peroxidase-labelled antibodies were detected using SuperSignal West Femto (Life technologies) with an image analyzer (LAS-4000; Fuji Photo Film). The bands that were evaluated by apparent molecular size were quantified using the ImageJ 1.47 h software program (NIH).

### Immunohistochemistry

All mice were deeply anaesthetized with sodium pentobarbital (200 mg kg^−1^, i.p.) and transcardially perfused with PBS, followed by 4% paraformaldehyde. Transverse spinal sections (40 μm, free floating) were incubated with an anti-KCNMB3 mouse monoclonal antibody (1:2,000) and anti-Iba1 rabbit polyclonal antibody (1:10,000; Wako, 019–19741), anti-GFAP rabbit polyclonal antibody (1:5,000; Dako, Z0334) or mature interleukin-1β (Santa Cruz, 1:1,000) for 5 days at 4 °C, followed by incubation with Cy3- or Alexa488-conjugated secondary antibodies (1:400; Jackson ImmunoResearch Laboratories). Some sections were incubated with an anti-NeuN monoclonal antibody Alexa Fluor 555 conjugate (1:2,000; Millipore, MAB377A5) for 2 days at 4 °C after treatment with the secondary antibody.

For the intracellular dye labelling in fixed slices, the transverse L4 spinal sections (100 μm, free floating) were incubated with anti-Iba1 (1:10,000) in PBS overnight at 4 °C. The spinal sections were incubated with rabbit secondary antibodies conjugated with Alexa Fluor 488 (1:400; Jackson ImmunoResearch Laboratories) for 2 h. Microelectrode filled with 2% Lucifer yellow in distilled water were inserted into Iba1-positive cells under the microscope equipped with a × 40 water-immersion objective (Carl Zeiss). The dye was injected into a cell by passing a −2 nA negative current. The spinal sections were refixed in 4% PFA, and then heated in PBS for 15 min at 90 °C to remove the anti-Iba1 antibody. The sections were incubated with an anti-Lucifer yellow rabbit polyclonal antibody (1:50,000; Life Technologies, A-5750) for 5 days at 4 °C. After being washed with PBS, the sections were incubated with rabbit secondary antibodies conjugated with Cy3 (1:400; Jackson ImmunoResearch Laboratories) and mounted in Vectashield (Vector Laboratories). The microglial morphology was captured using a C2si Confocal Laser Microscope (Nikon) and was analysed with ImageJ. Total length of microglial processes was measured using the Simple Neurite Tracer software program plug-in. Semi-automated tracing of process was performed for the 3D image data^66^.

### Patch clamp analyses

A whole-cell patch-clamp recording was made from the GFP-positive microglia or SR101-positive astrocyte located at lamina I of the L4 spinal cord in slice preparations^18^. The external solution consisted of (in mM) 117 NaCl, 3.6 KCl, 1.2 NaH_2_PO_4_, 1.2 MgCl_4_, 2.5 CaCl_2_, 11 glucose and 25 NaHCO_3_ saturated with 95% O_2_ and 5% CO_2_. Patch pipettes (8–10 MΩ) were filled with an internal solution containing the following solutions (in mM): 120 KCl, 2 MgCl_2_, 10 HEPES and 0.1 BAPTA adjusted to pH 7.3 with KOH. Voltage ramps from −120 to +30 mV were applied for 300 ms to induce BK currents. In some experiments, voltage ramp was started from −200 mV. The currents were measured at +30 mV and −200 mV. Free Ca^2+^ concentration of internal solution was calculated by MAXCHELATOR. BK currents in the lamina I astrocytes were extracted as the paxilline-sensitive component.

To record BK currents in lamina I neurons, patch pipettes (8–10 MΩ) were filled with an internal solution containing (in mM) 135 K-methanesulfonate, 10 NaCl, 10 HEPES, 4 ATP-Mg and 0.3 GTP-Na adjusted to pH 7.2 with Tris-base. The tail-currents were generated by 100-ms depolarization steps to 10 mV from a holding potential of −60 mV^67^. To record the mEPSCs, we performed the recording from lamina I spinal neurons. Patch pipettes (5–10 MΩ) were filled with an internal solution containing the following components (in mM): 135 potassium gluconate, 5 KCl, 0.5 CaCl_2_, 2 MgCl_2_, 5 EGTA, 5 HEPES and 5 ATP-Mg (ref. 68). The recordings were made at a holding potential of −70 mV in the presence of 1 μM tetrodotoxin, 10 μM bicuculline methiodide, a GABA_A_ receptor antagonist and 1 μM strychnine, a glycine receptor antagonist. Evoked currents were elicited by stimulation of the dorsal root entry. The second round of stimulation was delivered after an interval of 50 ms. The PPR was calculated as the amplitude ratio of the second to the first postsynaptic response after stimulation. For the current–intensity curve, root-evoked currents (*I*) were normalized to the maximum currents (*I*_Max_). The curve-fitting was examined using the GraphPad Prism 4 software program (GraphPad Software Inc., San Diego, CA, USA). A whole-cell patch clamp recording was made from MG6 was recorded at a holding potential of −60 mV (ref. 18). MG6 were treated with morphine (10 μM) for 24 h with or without naloxone or paxilline. ATP (3 mM), TNP-ATP (a P2X1, 2, 3, 4R inhibitor, 1 μM), PPADS (a P2X1, 2, 3, 5, 7R inhibitor, 10 μM), AA (1, 3, and 10 μM) and PGE_2_ (10 μM) were diluted with extracellular solution, and then applied to the cells. In some experiments, AA and PGE_2_ were mixed with the pipette solution.

### Ca^2+^ imaging

After 24 h of morphine treatment, MG6 and primary microglia were loaded with Fluo-4 AM (2.5 μM; Life Technologies) in the presence of pluronic F-127 (0.05%) for 10 min. In some experiments, *Slo1* gene-silenced MG6 and primary microglia were used. The cells were washed with a dye-free buffer for 30 min before experiments. Fluo-4 based images were obtained with a × 20 objective (0.75 NA) using the C2si Confocal Laser Microscope. The external solution consisted of (in mM) 140 NaCl, 1 KCl, 1 CaCl_2_, 10 HEPES, 1 MgCl_2_ and 10 glucose (pH 7.3). A Ca^2+^-free solution was prepared by replacing CaCl_2_ with 1 mM EGTA. The cells were incubated with thapsigargin (2 μM), a specific inhibitor of Ca^2+^-ATPases on the ER, for 30 min in Ca^2+^-free solution. Thapsigargin (1 μM), a specific inhibitor of Ca^2+^-ATPases on the endoplasmic reticulum, and nifedipine (10 μM), a voltage-gated Ca^2+^ channels blocker, were added in the external solution throughout the experiment. Paxilline (2 μM) or LaCl_3_ (50 μM) was diluted with external solution, and then applied to the microglia at a flow rate of 2.5 ml min^−1^. In some experiments, AA was applied to microglia without morphine stimulation. Images were captured at 0.5-s intervals. The fluorescence intensity (*F*) was measured by the NIS-Elements version AR Ver4.00.12 software program (Nikon). The results were presented as relative fluorescence values (Δ*F*/*F*_0_), where *F*_0_ stands for the fluorescence of controls (before the addition of Ca^2+^).

### Enzyme-linked immunosorbent assay

The Promega BDNF Emax ImmunoAssay System (Promega Co., Madison, WI) was used to measure the amount of BDNF in the supernatant of MG6 microglia according to the manufacturer's protocol. MG6 microglia was treated with morphine (10 μM) with or without naloxone (10 μM), paxilline (2 μM) and LaCl_3_ (50 μM) for 24 h, and then the supernatants were collected. Samples (100 μl) were run in triplicate on the plates.

### RT–PCR

For the semiquantitative reverse transcription–PCR (RT–PCR) analyses, RNA was extracted from the L4 dorsal spinal horns or the testes of mice following the i.t. injection of control siRNA or KCNMB3 siRNA using RNAiso Plus (Takarada, Japan) according to the manufacturer's instructions. RNA was also extracted from MG6 or primary microglia. A total of 1 μg of RNA was transcribed into complementary DNA (cDNA) using a QuantiTect Reverse Transcription Kit (Qiagen, Japan). An initial round of PCR was performed to amplify GAPDH. Each cDNA was amplified using primers listed in Supplementary Table 1.

For the single-cell PCR, GFP-labelled microglia in the lamina I of spinal cord slice were aspirated into patch pipettes with a tip diameter of 5–10 μm under a microscope. Pipettes with attached cells were broken into tubes containing 1.5 μl of 15 U RNasin Plus (Promega) and 2.5 μl of 25 mM DTT. The following reagents were mixed: 1 μl of oligo(dT)_20_, 1 μl of Random Hexamers (Roche), 1 μl of 10 mM dNTP and 2 μl of lysis buffer (10 mM Tris-HCl, 0.2% Nonidet-P40 and 50 mM guanidine thiocyanate). After mixing, the tubes were spun briefly, then heated for 5 min at 70 °C and cooled at least 2 min on ice. A total of 6 μl of 5 × RT Buffer, 1 μl of RNaseOUT, 1 μl of 200 U μl^−1^ SuperScript III, 1 μl of 0.1 M DTT and 1 μl of water were added and mixed, and the tubes were incubated at 50 °C for 50 min and 85 °C for 5 min. The single-cell cDNA products were used in a separate PCR. The primer sequences were listed in Supplementary Table 1. Platinum Taq polymerase (Life Technologies) was used for the amplification according to the manufacturer's protocol. The spinal cord cDNA was used as a positive control. The PCR samples were electrophoresed on 8% polyacrylamide gels to analyse the products.

### Statistical analyses

No statistical methods were used to predetermine sample sizes, but the sample sizes used are similar to those generally employed in the field. The data were collected and processed randomly. No data points were removed from statistical analysis. Genotype selection, drug administration, behavioural test and statistical analyses were separately and blindly conducted. All data are shown as the means±s.e.m. Data distribution was assumed to be normal but this was not formally tested. The statistical analyses were performed using a one-way analysis of variance (ANOVA) followed by Tukey's or Dunnett's *post hoc* test, a two-way ANOVA followed by the Bonferroni *post hoc* test, an unpaired *t*-test or a paired *t*-test using the GraphPad Prism 7 software program. A two-tailed Spearman's test was used to compare correlations. Unless otherwise indicated, the data met the assumptions of equal variances. Differences were considered to be significant for values at *P*<0.05.

### Data availability

The authors confirm that all relevant data are included in the article and its Supplementary Information Files.

## Additional information

**How to cite this article:** Hayashi, Y. *et al*. BK channels in microglia are required for morphine-induced hyperalgesia. *Nat. Commun.* 7:11697 doi: 10.1038/ncomms11697 (2016).

## Supplementary Material

Supplementary Information Supplementary Figures 1-22 and Supplementary Table 1
