# Supplementary material for: BK channels in microglia are required for morphine-induced hyperalgesia
Source: Nat Commun. 2016 May 31;7:11697. doi: 10.1038/ncomms11697 (PMC4895018; doi:10.1038/ncomms11697)
Supplement: Supplementary Information — Supplementary Figures 1-22 and Supplementary Table 1 [file ncomms11697-s1.pdf]

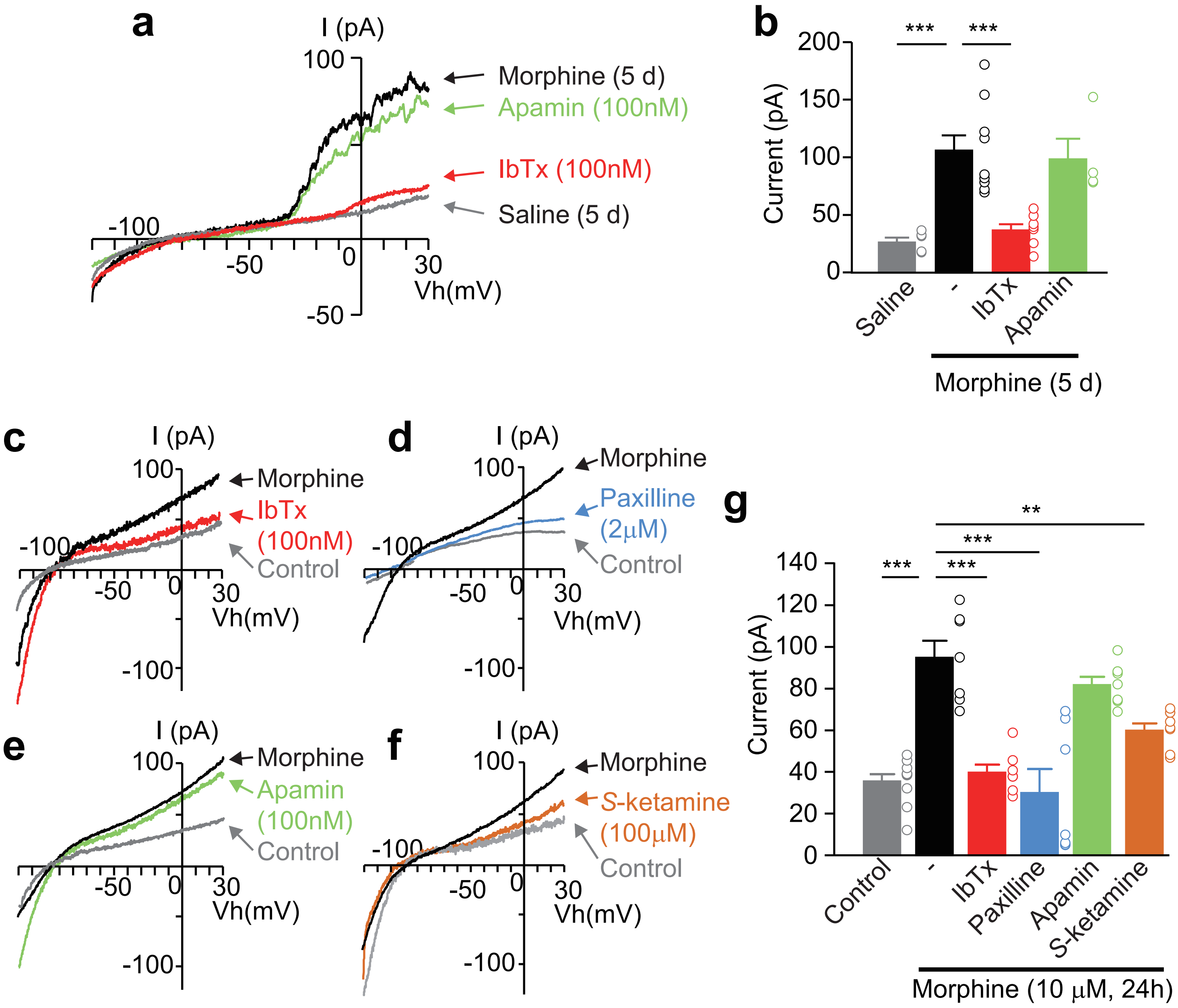

Supplementary Figure 1. Pharmacological properties of morphine-induced outward currents in the microglia. (**a**) Typical traces recorded from the lamina I spinal microglia after 5-days of saline or morphine (10 mg kg^{-1}) administration. IbTx (100 nM) and apamin (100 nM) were topically applied onto the lamina I spinal microglia from morphine-treated mice. (**b**) The amplitudes of the outward currents at +30 mV in the lamina I spinal microglia (n=5 from three mice for Saline, n=9 from four mice for Morphine, n=7 from three mice for IbTx, n=5 from three mice for apamin). (**c-f**) The outward currents recorded from MG6 after 24 h of morphine (10 μM) stimulation. IbTx (100 nM), paxilline (2 μM), apamin (100 nM) or *S*-ketamine (100 μM) were topically applied onto MG6. (**g**) The average amplitudes of the outward currents at +30 mV in MG6 (n=7 each). The data represent the means ± SEM. ****P*=0.0001, 0.0001 (each column), $F_{3, 27}$=36.56, a one-way ANOVA followed by the Tukey’ s *post-hoc* test (**b**). ****P*=0.0001 (Control versus Morphine), 0.0001 (Morphine versus Morphine+IbTx), 0.0001 (Morphine versus Morphine+Paxilline), ***P*=0.0048 (Morphine versus Morphine+*S*-ketamine), $F_{5, 39}$=18.35, a one-way ANOVA followed by the Tukey’ s *post-hoc* test (**g**).

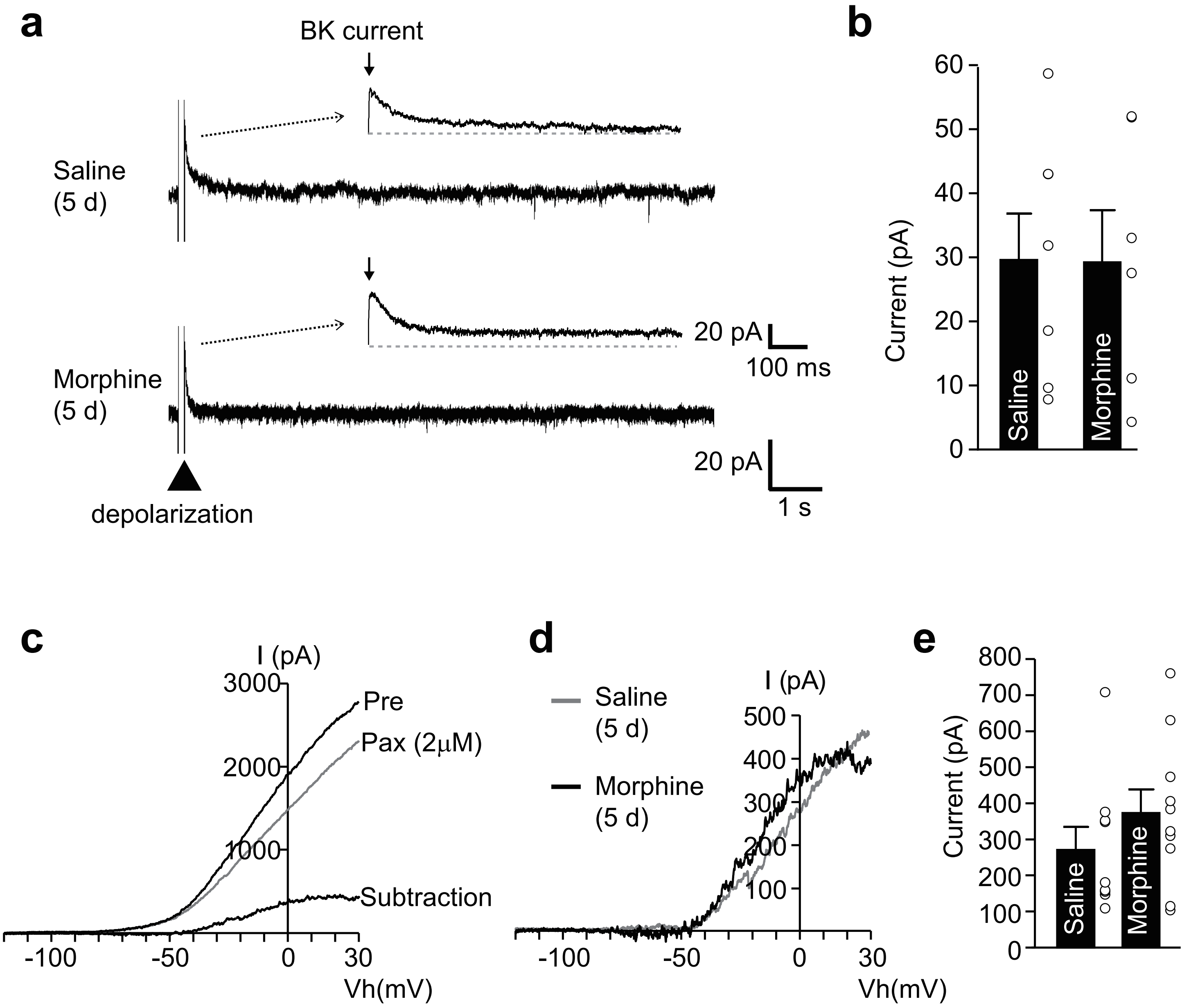

Supplementary Figure 2. BK currents in neurons and astrocytes in the lamina I spinal cord after five days after starting saline or morphine administration. (**a**) BK currents were elicited by a depolarization pulse of -60→+10 mV for a 100 ms duration (arrowhead). Broken arrows and lines indicate enlarged traces of BK currents (arrow) and basal current, respectively. (**b**) The average peak of BK currents in lamina I spinal neurons. (n=7 for Saline, n=6 for Morphine, from three mice each). The data represent the means ± SEM. (**c**) BK currents from SR101-positive astrocytes in the lamina I spinal cord were extracted as the paxilline-sensitive component. (**d**) Typical traces of BK currents in the lamina I spinal astrocytes. (**e**) The average amplitudes of BK currents at +30 mV in the lamina I spinal astrocytes (n=9 for Saline, n=10 for Morphine, from three mice each). The data were analyzed by an unpaired *t*-test (**b, e**). *P*=0.9733 (**b**) and *P*=0.7226 (**e**). t(11)=0.03429 (**b**), t (17)=1.114 (**e**)

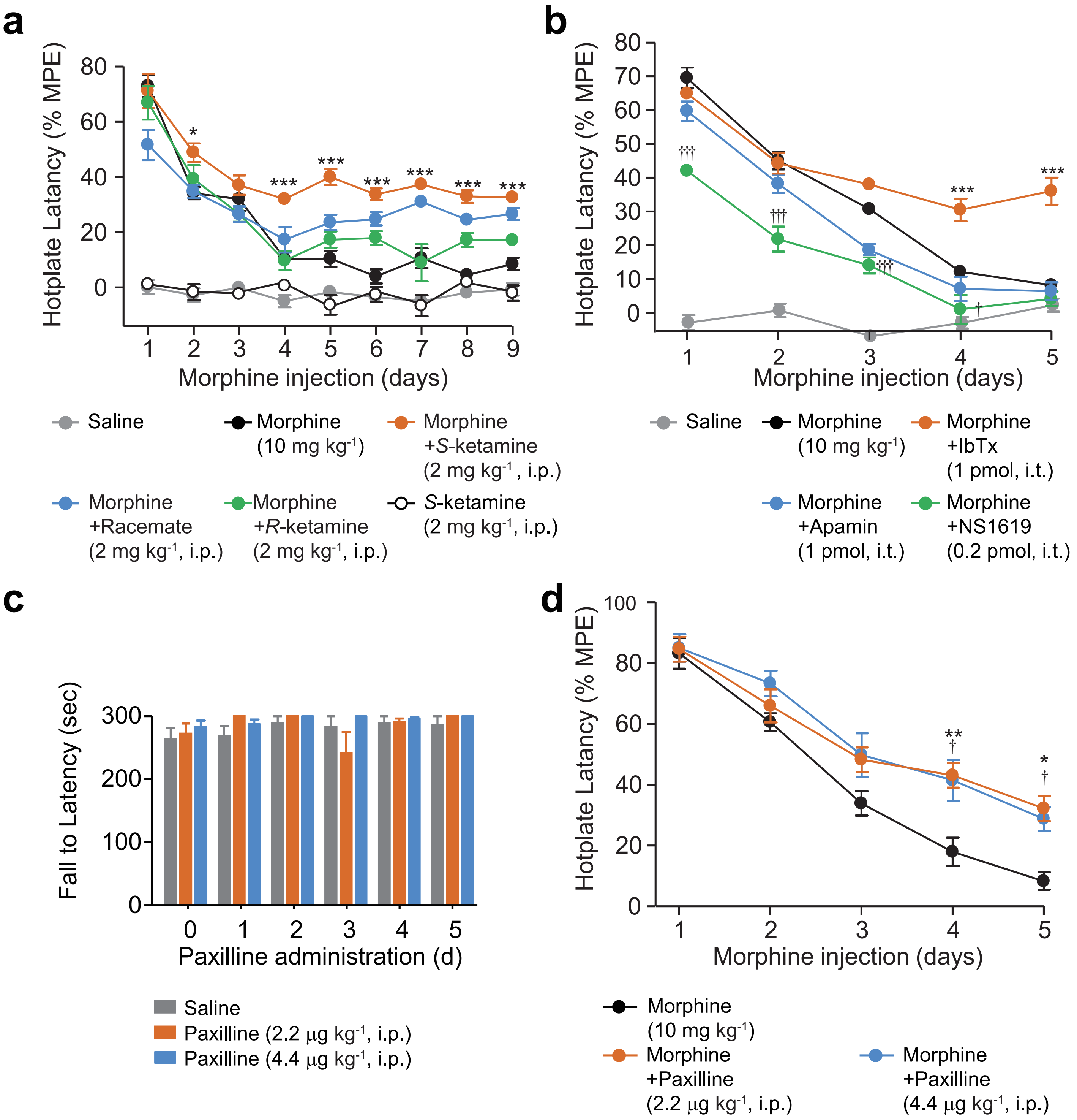

Supplementary Figure 3. Attenuation of the antinociceptive tolerance by supplementation with S-ketamine or paxilline. (**a**) Effects of each drugs on the analgesic response to morphine (n=9 each). (**b**) IbTx (1 pmol), apamin (1 pmol) or NS1619 (0.2 pmol) was intrathecally injected 30 min prior to morphine administration (n=9 each). (**c**) Motor performance was measured by fall latency on rotarod after 30 min of saline or paxilline (2.2 or 4.4 μg kg^{-1}) administration. (n=3 for Saline, n=4 for Paxilline (2.2 μg kg^{-1}), n=4 for Paxilline (4.4 μg kg^{-1}). (**d**) Paxilline (2.2 or 4.4 μg kg^{-1}) was intraperitoneally (i.p.) injected 30 min prior to morphine administration (n=7 for

Morphine, n=9 for Morphine+Paxilline (2.2 μg kg^{-1}), n=9 for Morphine+Paxilline (4.4 μg kg^{-1}). The data represent the mean ± SEM. *P=0.0114, ***P=0.0001, 0.0001, 0.0001, 0.0001, 0.0001, 0.0001 (Morphine versus Morphine+S-ketamine), (drug × time point interaction):$F_{5,432}$=285.6, a two-way ANOVA followed by the Bonferroni *post-hoc* test (**a**). ****P*=0.0001, 0.0001 (Morphine versus Morphine+IbTx), (drug × time point interaction):$F_{4,200}$=232.8, a two-way ANOVA followed by the Bonferroni *post-hoc* test (**b**). (drug × time point interaction):$F_{2, 48}$=2.22, a two-way ANOVA followed by the Bonferroni *post-hoc* test (**c**). **P*=0.0111, ***P*=0.0061 (Morphine versus Morphine+ Paxilline (2.2 μg kg^{-1}), †*P*=0.0132, 0.0193 (Morphine versus Morphine+ Paxilline (4.4 μg kg^{-1}), (drug × time point interaction):$F_{2,110}$=13.82, a two-way ANOVA followed by the Bonferroni *post-hoc* test (**d**).

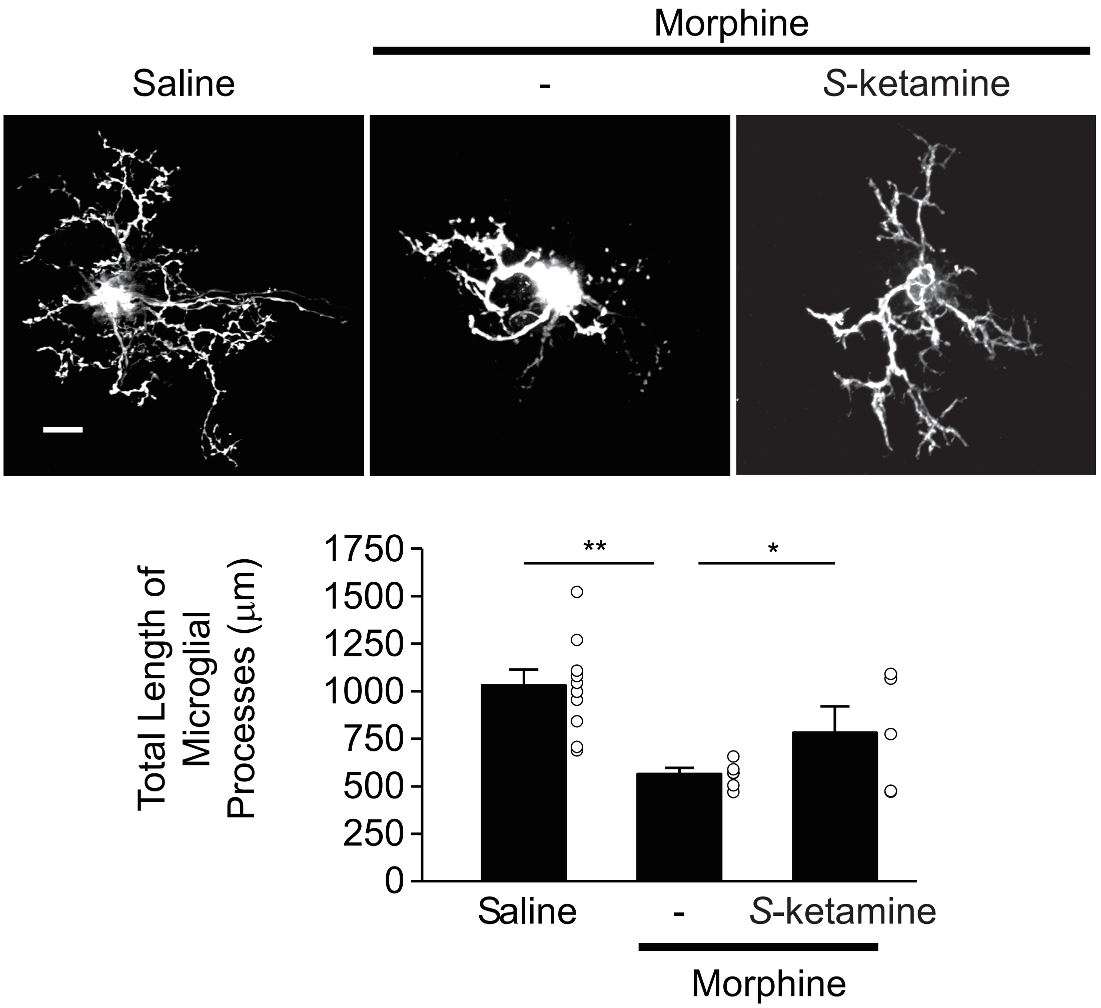

Supplementary Figure 4. Morphological changes in the spinal microglia following chronic morphine administration. Mice were sacrificed after 5-days of saline or morphine (10 mg kg^{-1}) administration. *S*-ketamine (2 mg kg^{-1}) was administered 30 min prior to morphine administration. Lucifer Yellow was injected into weakly stained Iba1-positive cells in the lamina I spinal cord. Thereafter microglia were visualized by an anti-Lucifer Yellow antibody. The total length of Z-stack images of microglia that were captured at 1-μm intervals was measured by 3D tracing with the ImageJ software program (n=10 for Saline, n=6 for Morphine, n=6 for Morphine+*S*-ketamine, from three mice each, **P*=0.0117, ***P*=0.0011, $F_{2, 18}$ =7.684, a one-way ANOVA followed by the Tukey’ s *post hoc* test). The data represent the means ± SEM. Scale bar, 50 μm.

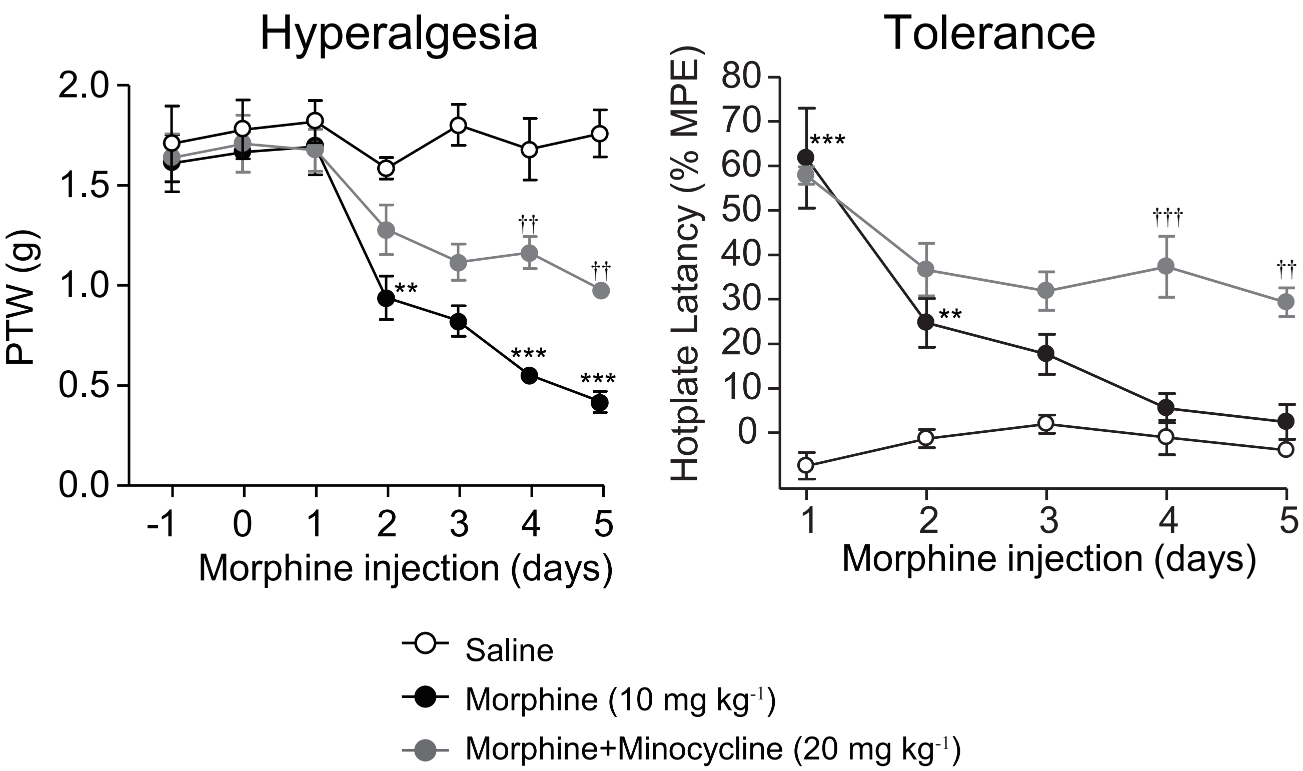

Supplementary Figure 5. Inhibitory effects of minocycline on MIH and antinociceptive tolerance. Minocycline (20 mg kg^{-1}) was intraperitoneally administered 30 min prior to morphine (10 mg kg^{-1}) administration. (n=5 for Saline, n=5 for Morphine, n=6 for Morphine+Minocycline). The data represent the mean ± SEM. ***P*=0.0034, ****P*=0.0001, 0.0001 (Saline versus Morphine), ††*P*=0.0041, 0.0092 (Morphine versus Morphine+Minocycline), (drug × time point interaction):$F_{2, 91}$=52.51 (left), a two-way ANOVA followed by the Bonferroni *post-hoc* test. ***P*=0.0022, ****P*=0.0001 (Saline versus Morphine), ††*P*=0.0016, †††*P*=0.0002 (Morphine versus Morphine+Minocycline), (drug × time point interaction):$F_{2, 60}$=90.61 (right), a two-way ANOVA followed by the Bonferroni *post-hoc* test.

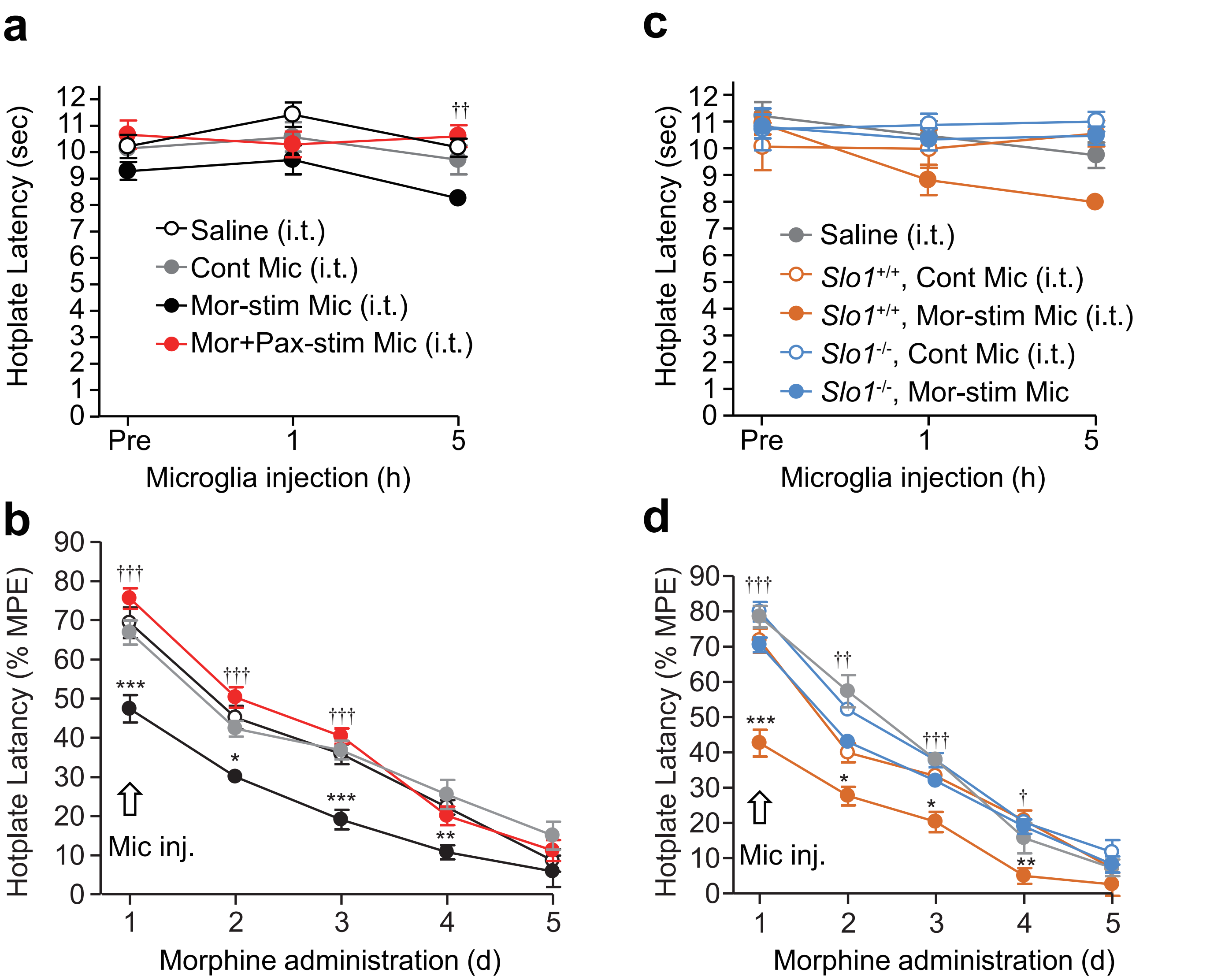

Supplementary Figure 6. Possible involvement of the microglial BK channels of the development of antinociceptive tolerance. (**a, c**) The time course of hotplate latency following intrathecal injection of morphine-stimulated primary microglia whose BK channels were pharmacologically (**a**) or genetically (**c**) silenced. Cont mic: non-stimulated primary microglia, Mor-stim mic: Morphine (10 μM)-stimulated primary microglia, Mor+Pax-stim mic: Morphine+Paxilline (2 μM)-stimulated primary microglia (n=9 each). (**b, d**) Effects of i.t. injection of morphine-stimulated primary microglia on morphine antinociceptive tolerance (n=9 each). The open arrow indicates the i.t. injection of primary microglia. The data were analyzed by a two-way ANOVA followed by the Bonferroni *post hoc* test. The data represent the mean ± SEM. ††*P*=0.0059 (Mor-stim Mic versus Mor+Pax-stim Mic), (drug × time point interaction): $F_{3, 93}$=7.36, a two-way ANOVA followed (**a**). ****P*=0.0001, **P*=0.0409, ****P*=0.0005, ***P*=0.0087 (Saline versus Mor-stim Mic), †††*P*=0.0001, 0.0001, 0.0001 (Mor-stim Mic versus Mor+Pax-stim Mic), (drug × time point interaction): $F_{3, 160}$=37.02, a two-way ANOVA (**b**). (drug × time point interaction):$F_{4, 120}$=4.1, a two-way ANOVA (**c**). ****P*=0.0001, **P*=0.0409, 0.0372, ***P*=0.0023 ($Slo1^{+/+}$ Cont Mic versus $Slo1^{+/+}$ Mor-stim Mic), †††*P*=0.0001, 0.0036, 0.0004, †*P*=0.0145 ($Slo1^{+/+}$ Mor-stim Mic versus $Slo1^{-/-}$ Mor-stim Mic), (drug × time point interaction): $F_{4, 200}$=47.97, a two-way ANOVA (**d**).

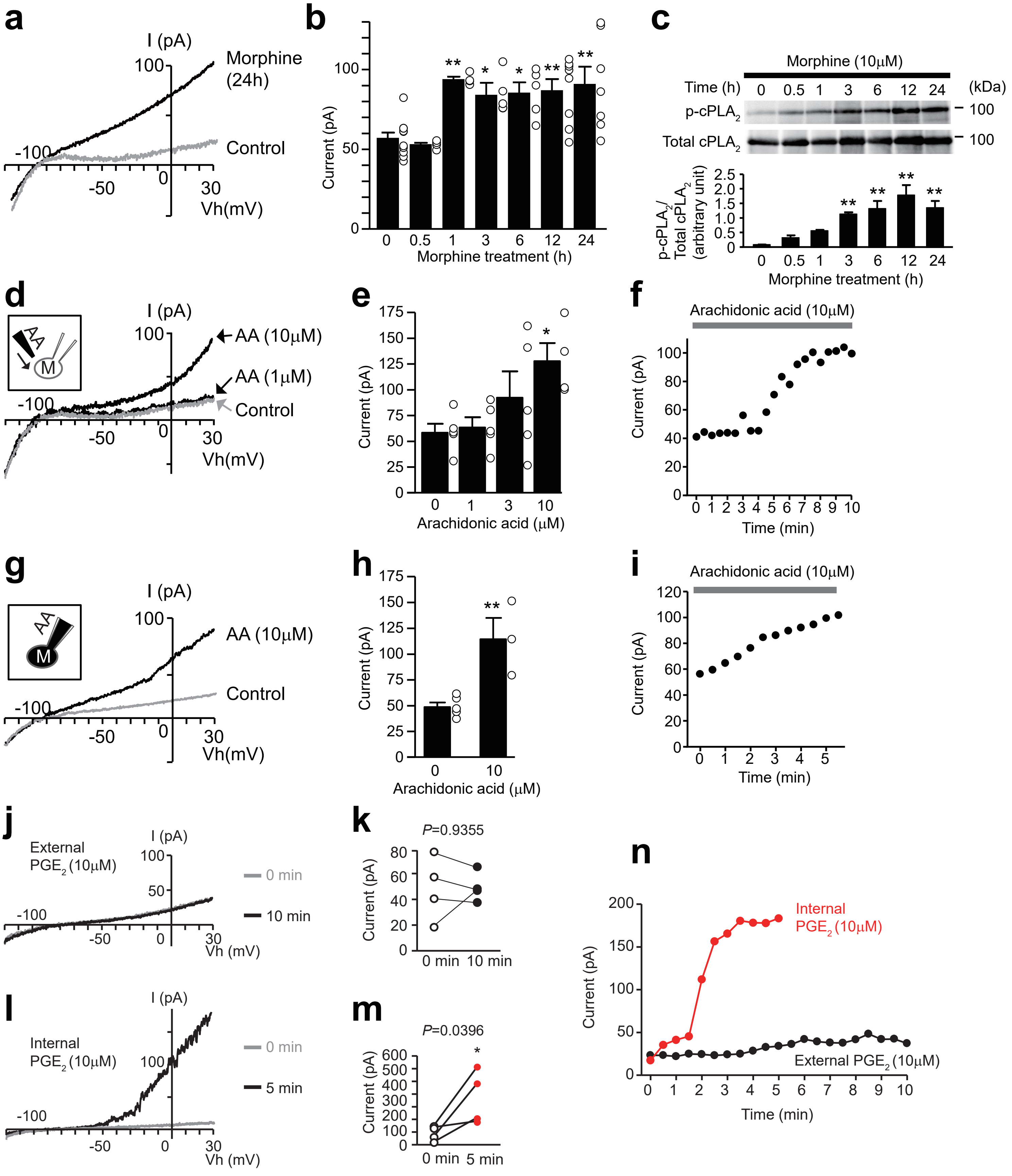

a
I (pA)
100
Morphine (24h)
Control
-100
-50
0
30
Vh(mV)
b
100
50
0
Current (pA)
** * * ** **
0 0.5 1 3 6 12 24
Morphine treatment (h)
c
Morphine (10μM)
Time (h) 0 0.5 1 3 6 12 24 (kDa)
p-cPLA2
Total cPLA2
100
100
p-cPLA2/ Total cPLA2 (arbitrary unit)
2.5 2.0 1.5 1.0 0.5 0
** ** ** **
0 0.5 1 3 6 12 24
Morphine treatment (h)
d
AA
M
I (pA)
100
AA (10μM)
AA (1μM)
Control
-100
-50
0
30
Vh(mV)
e
175 150 125 100 75 50 25 0
Current (pA)
*
0 1 3 10
Arachidonic acid (μM)
f
Arachidonic acid (10μM)
100 80 60 40 20 0
Current (pA)
0 1 2 3 4 5 6 7 8 9 10
Time (min)
g
AA
M
I (pA)
100
AA (10μM)
Control
-100
-50
0
30
Vh(mV)
h
175 150 125 100 75 50 25 0
Current (pA)
**
0 10
Arachidonic acid (μM)
i
Arachidonic acid (10μM)
120 100 80 60 40 20 0
Current (pA)
0 1 2 3 4 5
Time (min)
j
External PGE2 (10μM)
I (pA)
100
50
-100
-50
0
30
Vh (mV)
0 min
10 min
k
P=0.9355
80 60 40 20 0
Current (pA)
0 min 10 min
l
I (pA)
Internal PGE2 (10μM)
100
-100
-50
0
30
Vh (mV)
0 min
5 min
m
P=0.0396
*
600 500 400 300 200 100 0
Current (pA)
0 min 5 min
n
200 150 100 50 0
Current (pA)
Internal PGE2 (10μM)
External PGE2 (10μM)
0 1 2 3 4 5 6 7 8 9 10
Time (min)

Supplementary Figure 7. Direct activation of BK currents by arachidonic acid in microglia. (**a**) The currents activation of BK channels in MG6 after 24 h of morphine (10 μM) stimulation. (**b**) The time course of BK currents at +30 mV after morphine stimulation (n=10/4/4/4/5/8/7 cells). (**c**) The time course of the phosphorylation of p-cPLA$_2$ and the ratio of p-cPLA$_2$/cPLA$_2$ in MG6 after morphine stimulation. (**d-i**) The exogenous application of arachidonic acid (AA) potentiated BK currents in MG6 . The inset is an illustration of the extracellular (**d**) or intracellular (**g**) perfusion of AA. n=5/5/5/4 cells in (**e**) and n=5/3 cells in (**h**). The traces show BK currents after 10 min of AA treatment (**d, g**). Concentration (**e, h**) and time (**f, i**)-dependent activation of BK currents after AA (10 μM) treatment. (**j-p**) The effects of extracellular (**j**) or intracellular (**l**) perfusion of prostaglandin E$_2$ (PGE$_2$) on BK currents in MG6. n=4 cells (**k, m**). The BK currents at +30 mV at 10 min (**k**) or 5 min (**m**) after PGE$_2$ treatment. (**n**) The time course of BK currents after PGE$_2$ treatment. The data represent the mean ± SEM. ***P*=0.0063, **P*=0.0456, 0.0285, ***P*=0.0056, 0.0024 (versus 0 h), $F_{6, 35}$=5.438, a one-way ANOVA followed by the Dunnett's *post hoc* test (**b**). ***P*=0.0099, 0.0035, ****P*=0.0002, ***P*=0.0028 (versus 0 h), $F_{6, 14}$=9.474, a one-way ANOVA followed by the Dunnett's *post hoc* test (**c**). **P*=0.0327 (versus 0 h), $F_{3, 15}$ =3.335, a one-way ANOVA followed by the Dunnett's *post hoc* test (**e**). ***P*=0.0069 (versus 0 h), t(6)=4.033, a paired *t*-test (**h**). *P*=0.9355, t(6)=0.08436 , a paired *t*-test (**k**). **P*=0.0396, t(6)=2.619, a paired *t*-test (**m**).

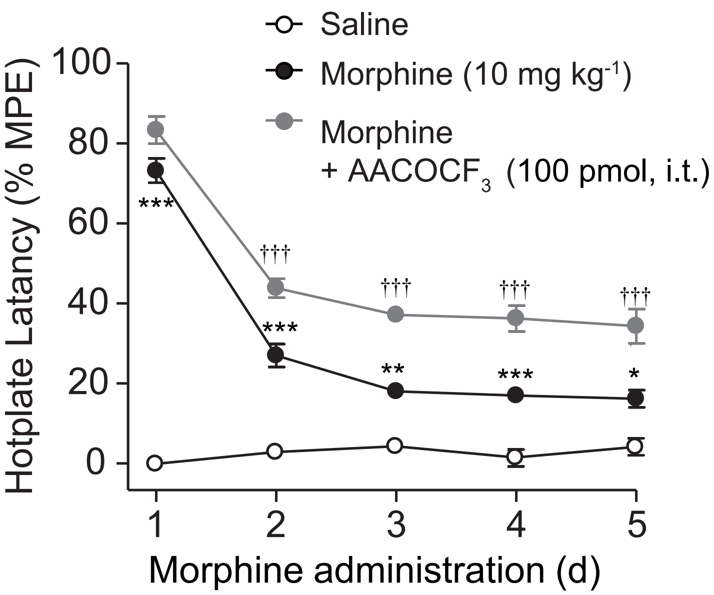

Supplementary Figure 8. Supplementation with the PLA_2 inhibitor had inhibitory effects on antinociceptive tolerance. $AACOCF_3$ (10 μM) was intrathecally injected 30 min prior to morphine administration. The data were analyzed by a two-way ANOVA followed by the Bonferroni *post hoc* test. The data represent the means ± SEM. (n=5 for Saline, n=5 for Morphine, n=5 for Morphine + $AACOCF_3$). ****P*=0.0001, 0.0001, ***P*=0.0026, ****P*=0.0003, **P*=0.0101 Saline versus Morphine), †††*P*=0.0001, 0.0001, 0.0001, 0.0001 (Morphine versus Morphine+$AACOCF_3$), (drug × time point interaction): $F_{2, 60}$=378.77.

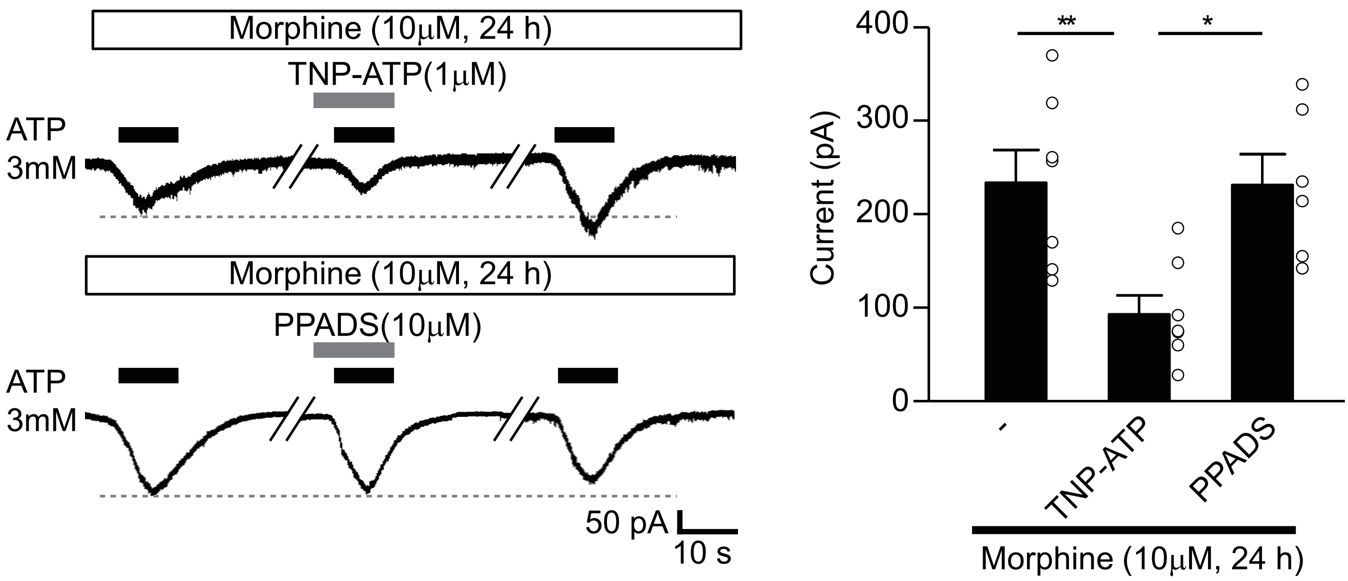

Supplementary Figure 9. Morphine potentiates P2X4 receptor-mediated ATP currents in MG6 microglia. ATP-induced currents in MG6 were recorded after 24 h of morphine (10 μM)-stimulation. Typical traces of ATP-induced inward currents. The responses recorded from the same cell throughout the whole-cell configuration. The slash between the traces indicate 3 min interval (n=7/6/7 cells). Black bars indicate ATP (3 mM) application. Gray bars indicate TNP-ATP (1 μM) and PPADS (10 μM) application, respectively. The data were analyzed by a one-way ANOVA followed by the Tukey' s *post hoc* test. The data represent the means ± SEM. **P*=0.0128, ***P*=0.0086 ($F_{2, 17}$=7.506, one-way ANOVA followed by the Tukey' s *post hoc* test). Scale bar, 50 pA and 10 s.

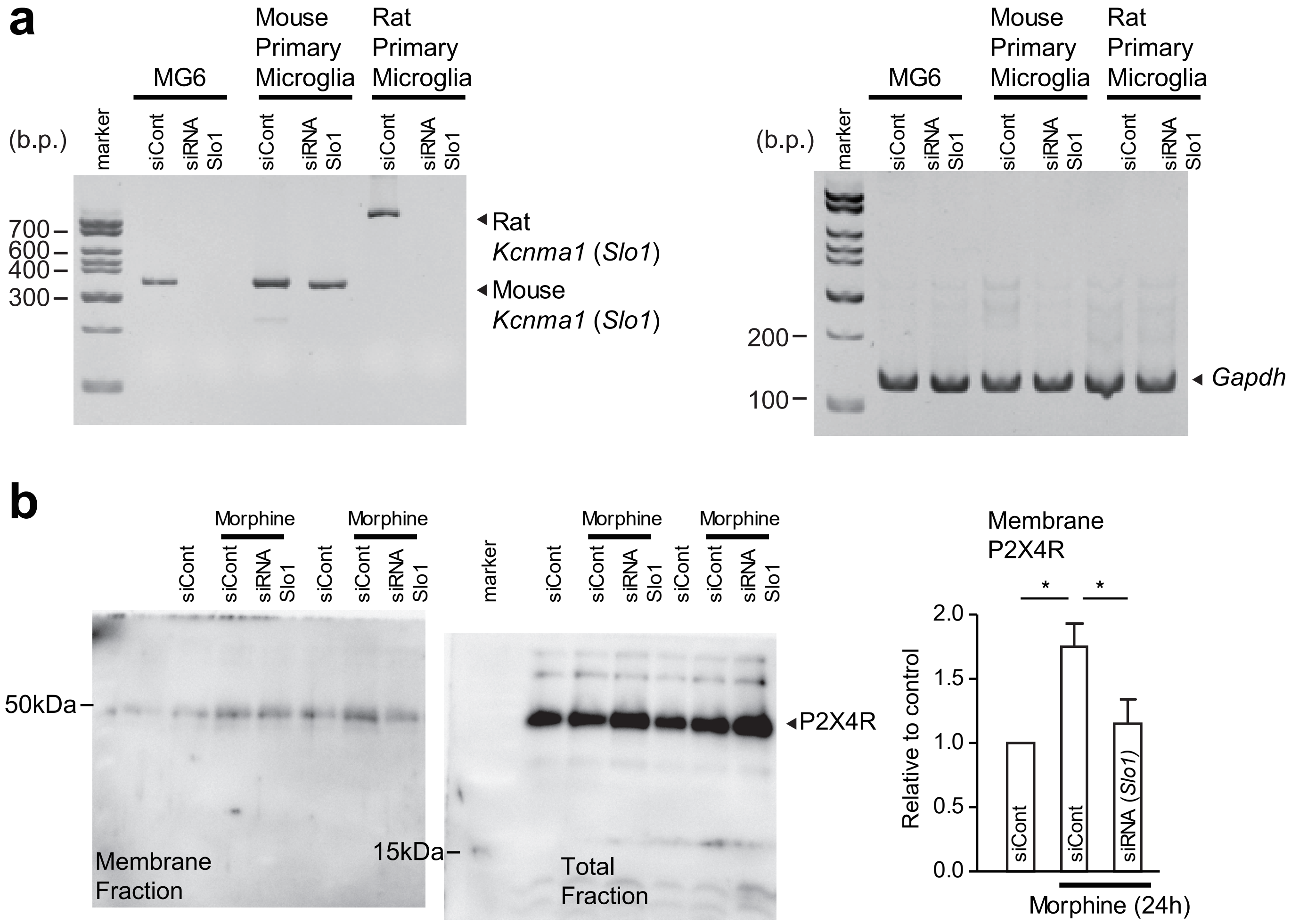

Supplementary Figure 10. Gene-silencing of Slo1 suppresses the morphine-induced increase in membrane P2X4Rs in microglia. (**a**) Northern blot of *Kcnma1* (*Slo1*) and *Gapdh* mRNA in MG6 and primary microglia. Gene-silencing of Slo1 was accomplished by siRNA transfection in microglia. Left panel indicate *Slo1* mRNA. The size of mouse *Slo1* and rat *Slo1* mRNA are 328 and 957 bp, respectively. Right panel indicate *Gapdh* mRNA. (**b**) Western blot of P2X4Rs in primary microglia. Slo1 siRNA inhibited morphine-induced increase in membrane P2X4Rs in primary microglia. The data were analyzed by a one-way ANOVA followed by the Tukey' s *post hoc* test. The data represent the means ± SEM. **P*=0.0160, 0.0473, $F_{2, 11}$=6.976.

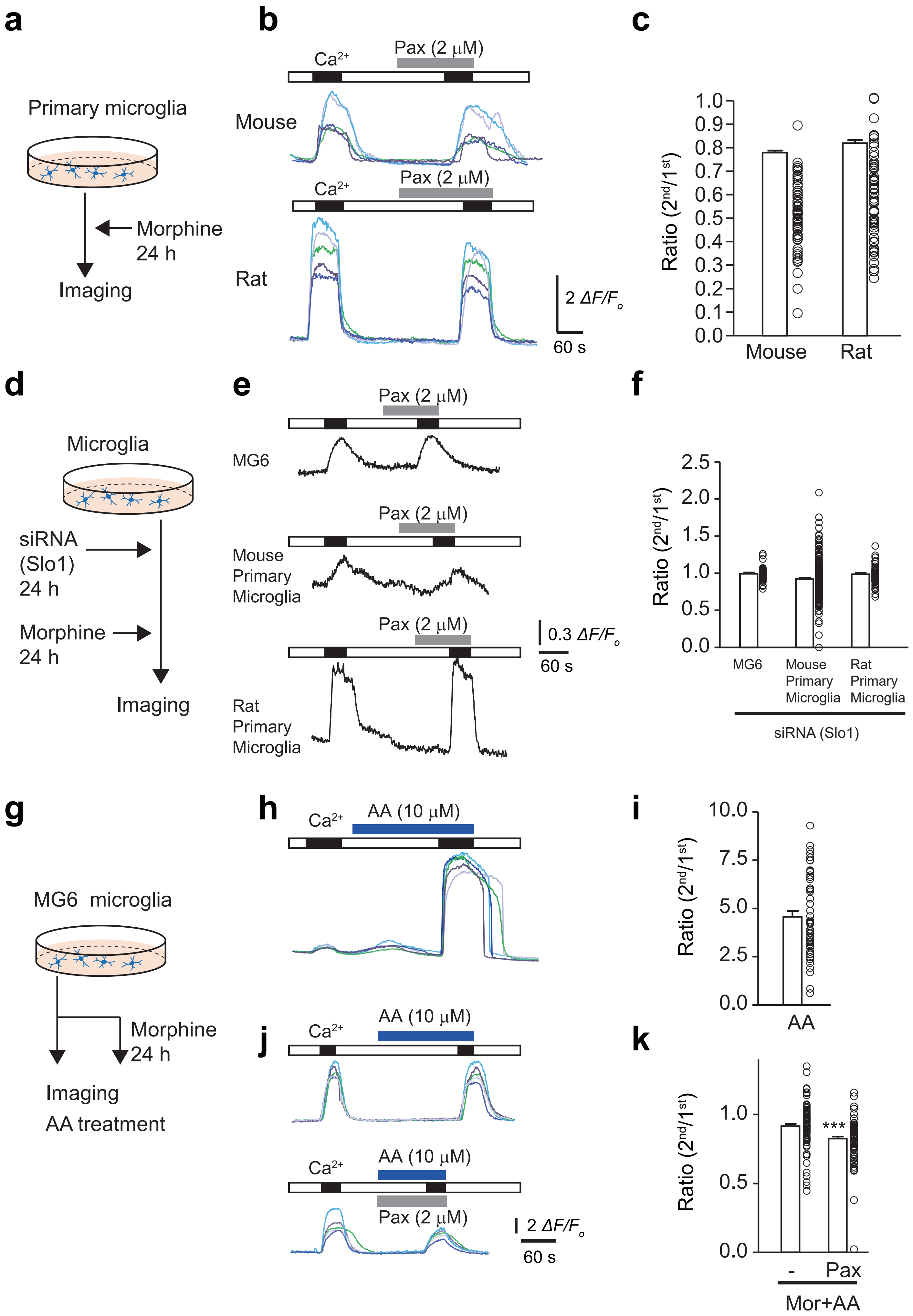
a
Primary microglia
Morphine
24 h
Imaging
b
Ca2+
Pax (2 μM)
Mouse
Rat
2 ΔF/Fo
60 s
c
Ratio (2nd/1st)
Mouse
Rat
d
Microglia
siRNA
(Slo1)
24 h
Morphine
24 h
Imaging
e
MG6
Mouse Primary Microglia
Rat Primary Microglia
0.3 ΔF/Fo
60 s
f
siRNA (Slo1)
g
MG6 microglia
Imaging
AA treatment
h
AA (10 μM)
i
AA
j
k
***
-
Pax
Mor+AA

Supplementary Figure 11. Involvement of BK channels on Ca^{2+} influx in primary mouse and rat microglia. (**a-c**) Paxilline attenuated Ca^{2+} influx in primary microglia. (d-e) Effects of *Slo1* gene-silencing on the Ca^{2+} influx. (**g-k**) Effects of arachidonic acid (AA) on the Ca^{2+} influx. Schematic illustration of time schedule of drug treatment on microglia (**a, d, g**). Typical traces of Ca^{2+} influx (**b, e, h, j**). The average ratio of 2nd/1st Ca^{2+} responses (**c, f, i, k**). n=65/60 cells (**c**), n=37/220/60 cells (**f**), n=50 cells (**i**), n=87/91 cells (**k**). The data represent the means ± SEM. ***P=0.0001, t (176)=3.941 (an unpaired *t*-test) (**k**).

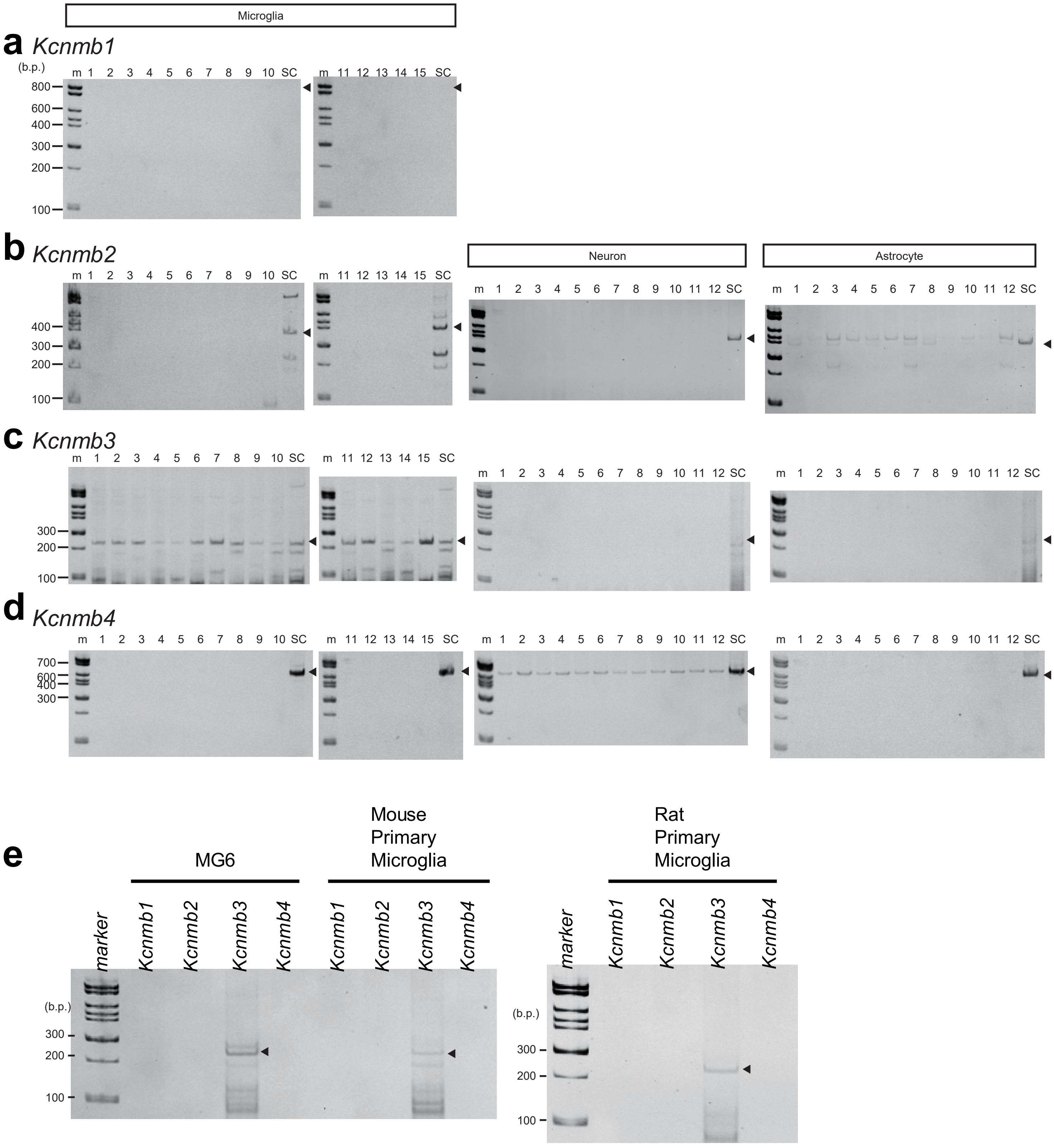
Microglia
a Kcnmb1
(b.p.)
m 1 2 3 4 5 6 7 8 9 10 SC
m 11 12 13 14 15 SC
800
600
400
300
200
100
b Kcnmb2
Neuron
Astrocyte
m 1 2 3 4 5 6 7 8 9 10 11 12 SC
400
300
200
100
c Kcnmb3
d Kcnmb4
700
600
400
300
e
MG6
Mouse Primary Microglia
Rat Primary Microglia
marker
Kcnmb1
Kcnmb2
Kcnmb3
Kcnmb4

Supplementary Figure 12. The microglia exclusively express the BK channel auxiliary KCNMB3 subunits. (**a-d**) The Northern blot analyses of *Kcnmb1* (**a**), *Kcnmb2* (**b**), *Kcnmb3* (**c**) and *Kcnmb4* (**d**) mRNA. Single cells (microglia, neurons and astrocytes) were collected from the lamina I spinal cord with a patch pipette. Subsequently, a single cell PCR was performed. m, marker. SC, spinal cord. The arrowheads indicate the target mRNA. n=12-15 cells from three mice each. (**e**) The Northern blot of *Kcnmb* mRNA in the MG6 and primary microglia. Each type of microglia expressed only *Kcnmb3* mRNA. The arrowheads indicate *Kcnmb3* mRNA.

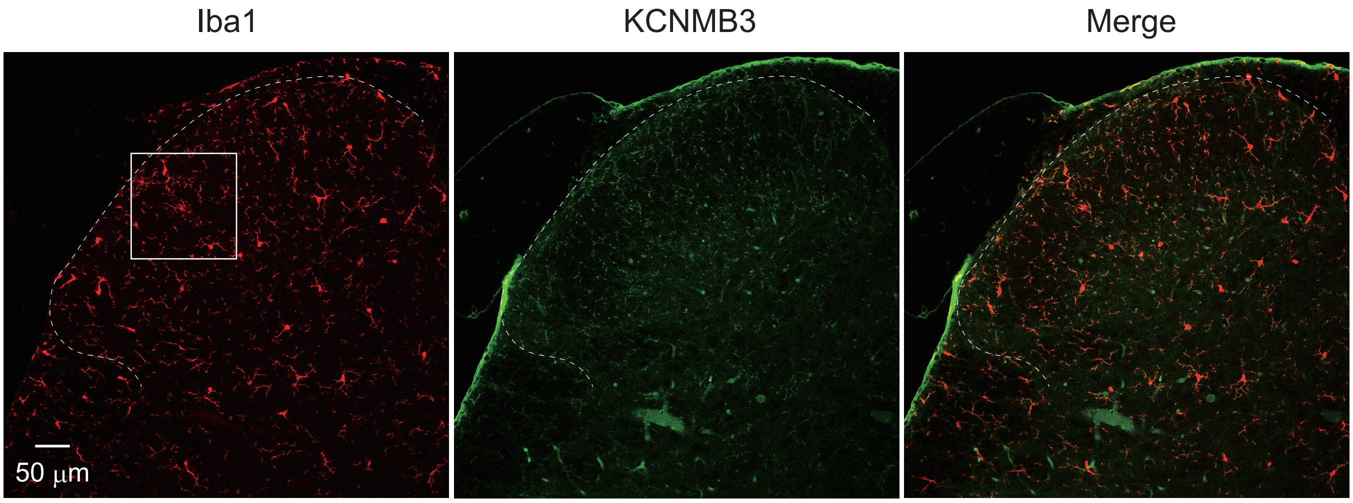

Supplementary Figure 13. Immunofluorescence of KCNMB3 in the dorsal spinal cord. Red and green indicate Iba1, a marker for microglia, and KCNMB3 immunofluorescence, respectively. Broken lines indicate the border of white and gray matter. Inset represents in Figure 7b.

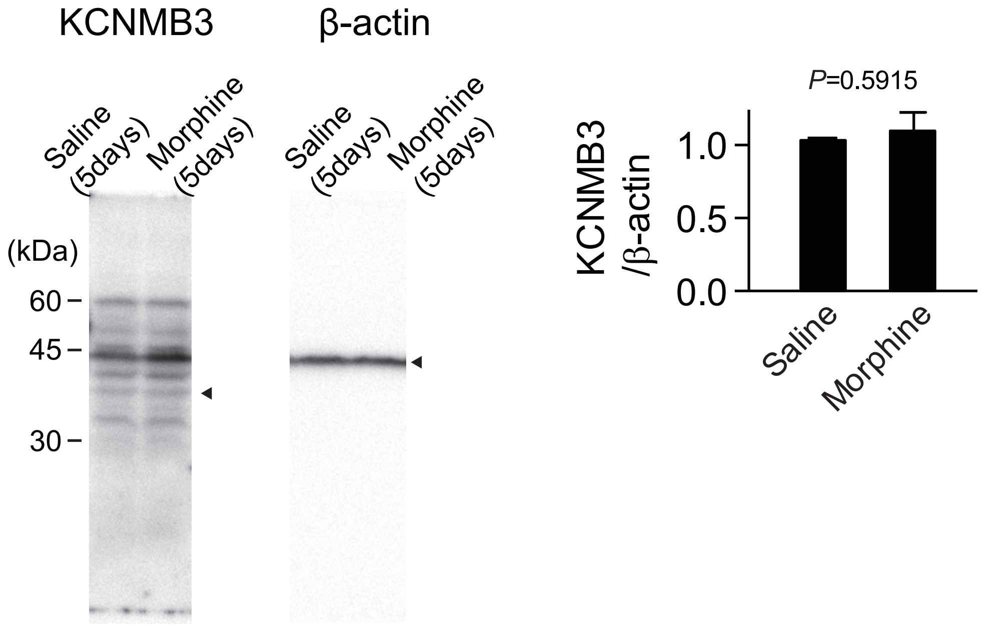

Supplementary Figure 14. The expression levels of KCNMB3 in the L4 spinal dorsal horn. The levels of KCNMB3 protein in the L4 spinal dorsal horn were not changed by the administration of morphine (10 mg kg^{-1}) for five days. The data were analyzed by an unpaired *t*-test. The data represent the means ± SEM. n=3 mice each, *P*=0.5915.

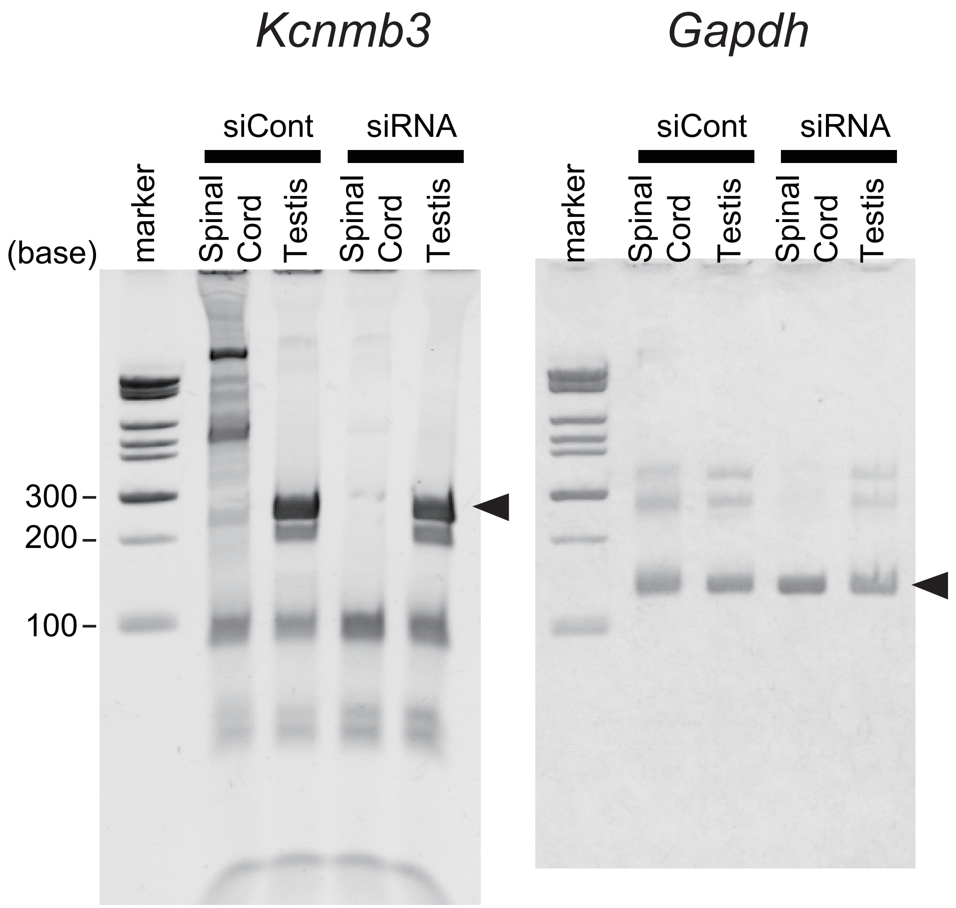

Supplementary Figure 15. The depletion of the Kcnmb3 gene in the L4 spinal dorsal horn after the i.t. injection of siRNA. Control siRNA (siCont) or KCNMB3 siRNA (#3) was intrathecally injected once daily for 4 days. The L4 spinal dorsal horn and testis were collected and northern blotting was performed. The Northern blot of *Kcnmb3* and *Gapdh* mRNA in the spinal cord and testis.

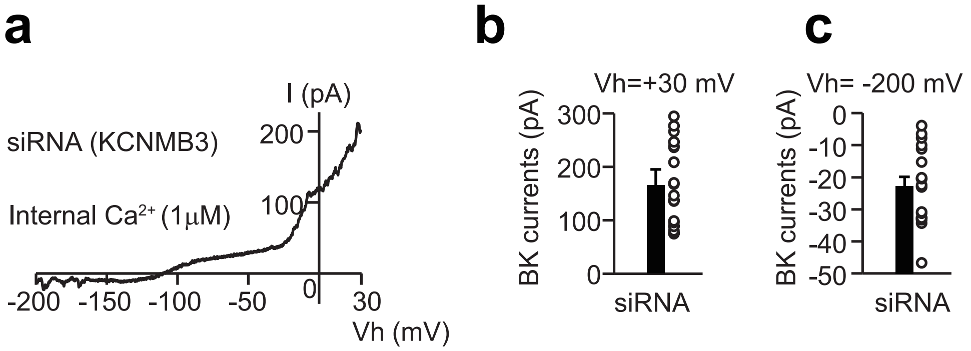

Supplementary Figure 16. The channel properties of KCNMB3 in the lamina I spinal microglia. (**a-c**) BK currents in the lamina I spinal microglia from *Kcnmb3* gene-silenced mice. The pipette solution contained 1 μM of Ca^{2+}. A ramp-pulse from -200 to +30 mV was applied for 300-msec. Typical traces of BK currents from the lamina I spinal microglia (**a, b**). The average of BK currents at +30 mV (**c**) and -200 mV (**d**). The data represent the means ± SEM. n=18 from three mice each.

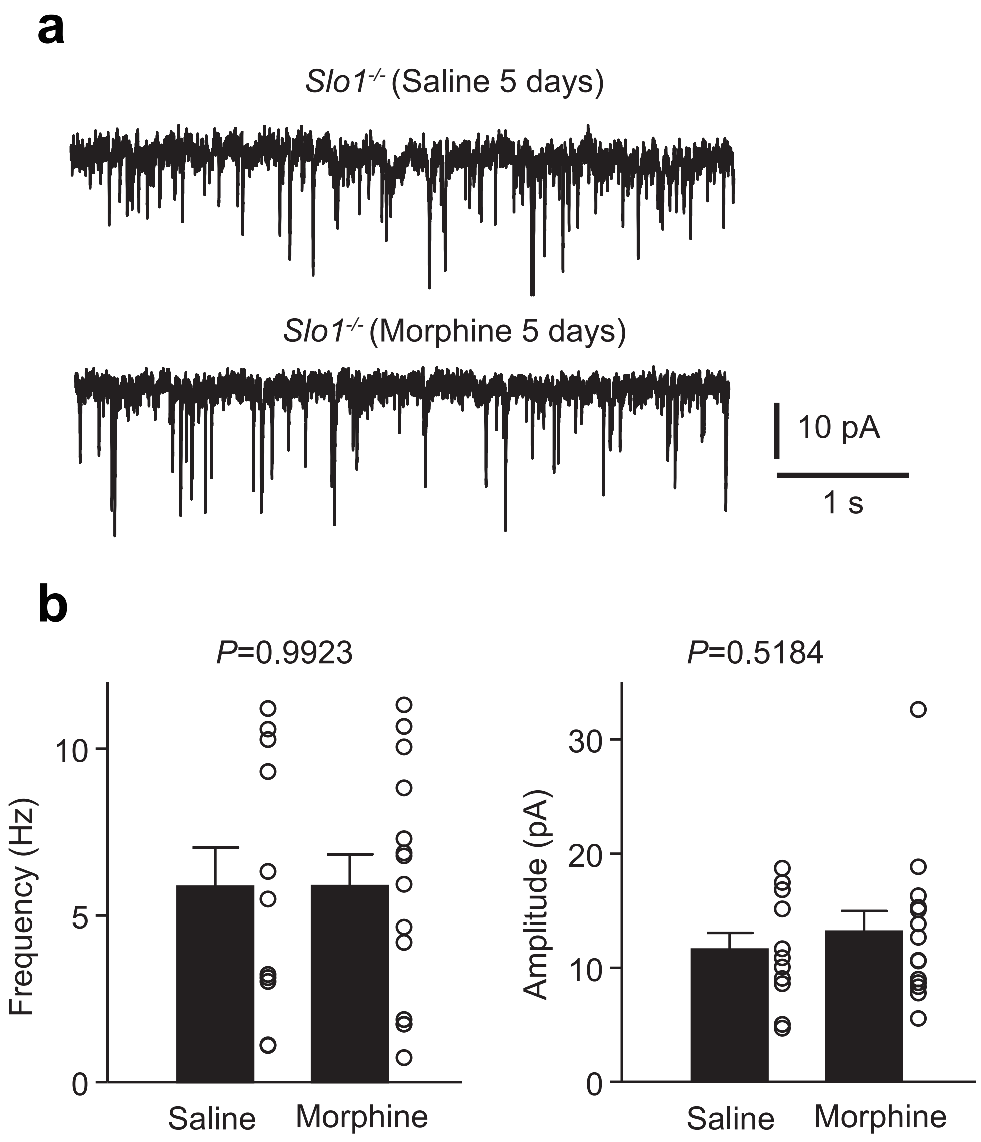

Supplementary Figure 17. Neurotransmission in the lamina I spinal neurons in *Slo1*$^{-/-}$ mice. (**a**) Typical traces of mEPSCs recorded from lamina I neurons. The recordings were performed from *Slo1*$^{-/-}$ mice after the 5-day administration of saline or morphine (10 mg kg^{-1}). (b) The averaged frequency and amplitude of the mEPSCs (n=11 from three mice for saline; n=14 from three mice for morphine). The data represent the means ± SEM (*P*=0.9923, *P*=0.5184, unpaired *t*-test).

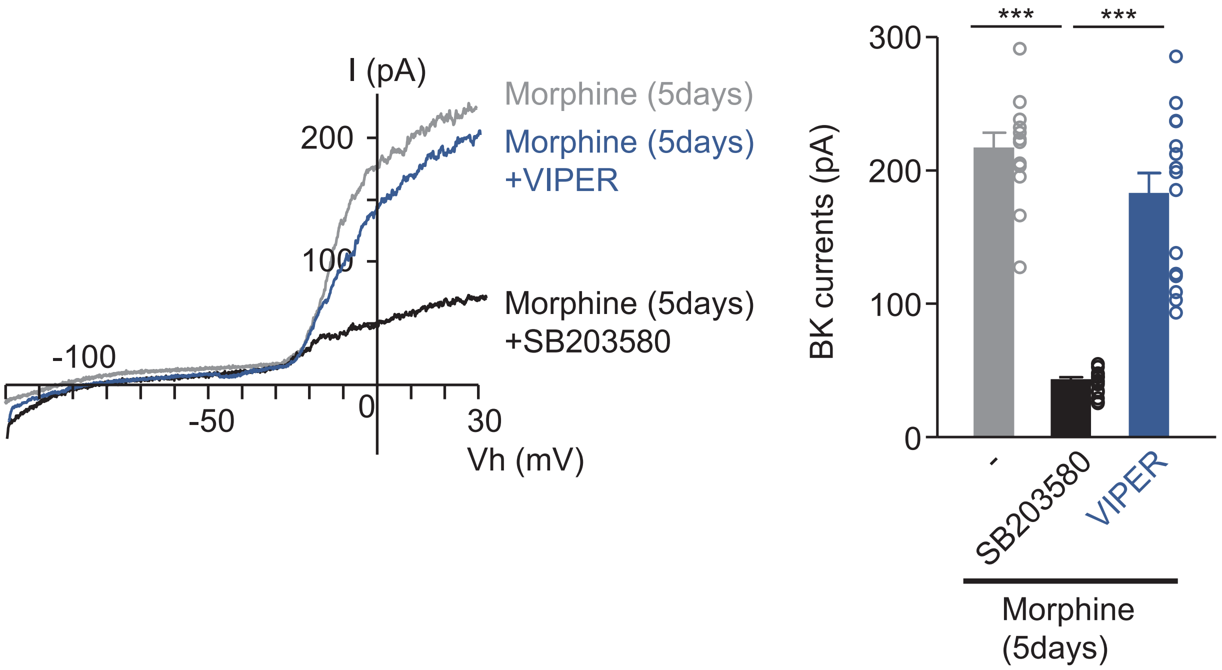

Supplementary Figure 18. The effects of the intrathecal administration of p38MAPK inhibitor or TLR4 inhibitor on morphine-induced BK channel activation in the lamina I spinal microglia. SB203580 (a p38MAPK inhibitor, 30 nmol 10 μL^{-1}) or VIPER (a TLR4 inhibitor, 10 nmol 10 μL^{-1}) were intrathecally injected 30 min before the administration of morphine (10 mg kg^{-1}). After 5-days administration (inhibitor and morphine), patch clamp analyses of the lamina I spinal microglia were performed. The left panel shows the typical traces of BK currents in the lamina I spinal microglia. The right panel shows the average BK currents in the lamina I spinal microglia at +30 mV. The data represent the means ± SEM (****P*=0.0001,0.0001, $F_{2, 41}$=72.69, a one-way ANOVA followed by Tukey' s *post hoc* test).

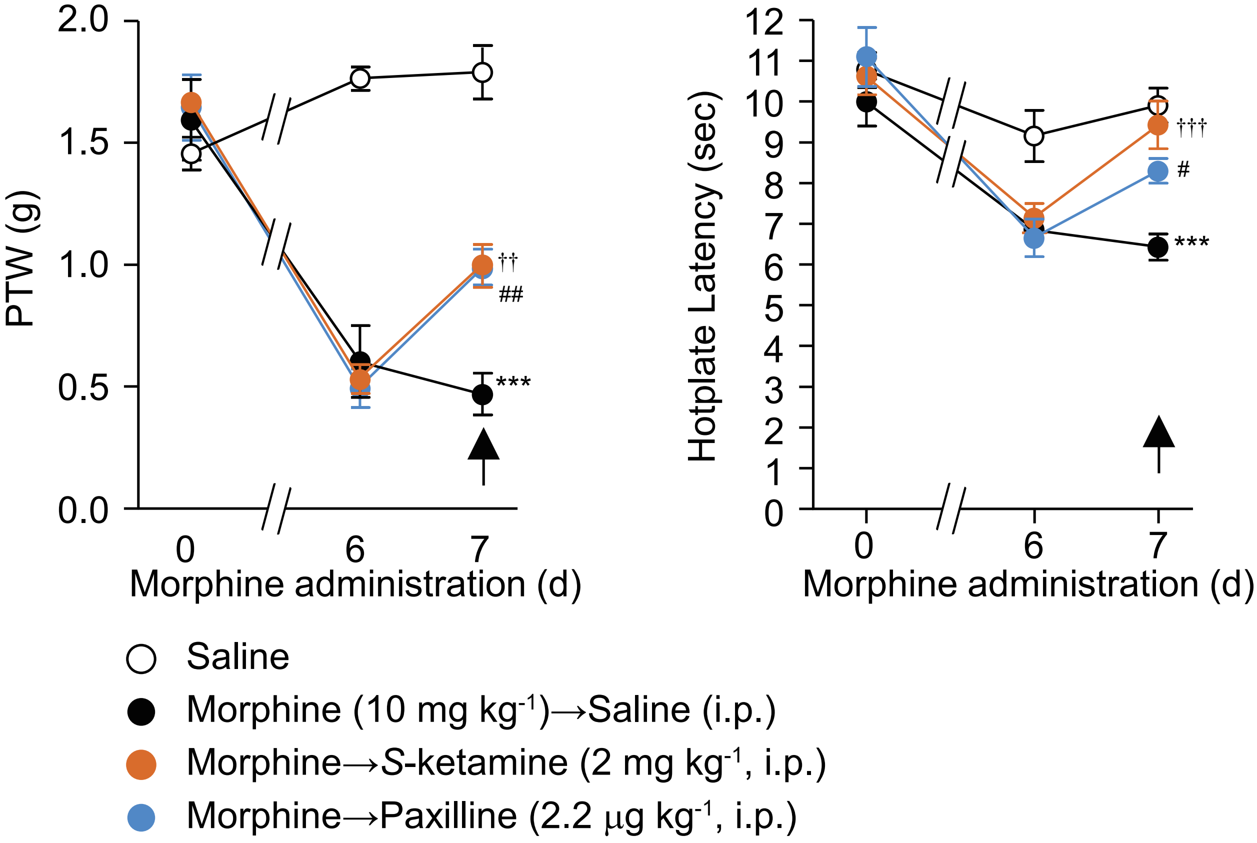

Supplementary Figure 19. The amelioration of established morphine-induced mechanical and heat hypersensitivity with a single injection of S-ketamine or paxilline. Morphine (10 mg kg^{-1}) was administered twice a day for 6-days. The following day, the mice were treated with saline, S-ketamine (2 mg kg^{-1}) or paxilline (2.2 μg kg^{-1}) for 60 min prior to a behavioral analysis. The arrow indicates the administration of the drug. The data were analyzed by a two-way ANOVA followed by the Bonferroni *post hoc* test. The data represent the means ± SEM. (n=8 for saline, n=5 for morphine→saline, n=5 for morphine→*S*-ketamine, n=5 for morphine→paxilline). ****P*=0.0001 (Saline versus Morphine→Saline), ††*P*=0.0031 (Morphine→Saline versus Morphine→*S*-ketamine), ##*P*=0.0043 (Morphine→Saline versus Morphine→ Paxilline), (drug × time point interaction): $F_{3, 57}$=46.13 (left). ****P*=0.0001 (Saline versus Morphine→Saline), †††*P*=0.0001 (Morphine→Saline versus Morphine→ *S*-ketamine), #*P*=0.0392 (Morphine→Saline versus Morphine→Paxilline), (drug × time point interaction): $F_{3, 60}$=10.33 (right).

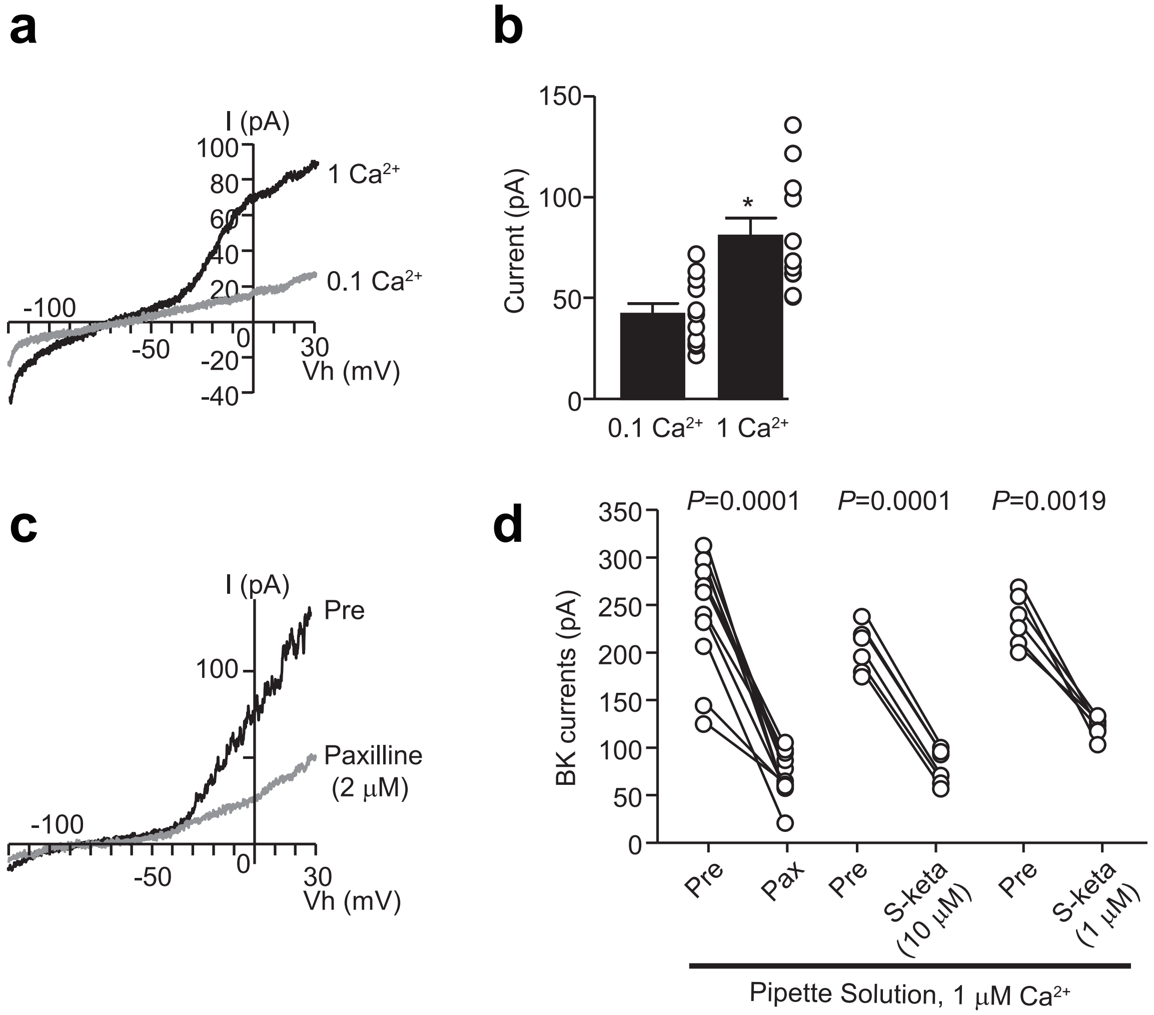

Supplementary Figure 20. The Ca^{2+} sensitivity of BK currents in the lamina I spinal microglia. (**a-d**) A ramp pulse-elicited BK currents in the lamina I spinal microglia from naïve mice. (a, b) The pipette solution contained 0.1 μM or 1 μM of Ca^{2+}. (**c, d**) The intracellular Ca^{2+} (1 μM)-elicited BK currents were sensitive to the bath application of paxilline (2 μM). (**d**) The bath application of *S*-ketamine (1 or 10 μM) significantly attenuated BK currents in the lamia I spinal microglia. (**b, d**) The average of BK currents at +30 mV in the lamina I spinal microglia. The data were analyzed by (**b**) an unpaired *t*-test and (**d**) a paired *t*-test. The data represent the means ± SEM. (**b**) n=11 from three mice each, **P*=0.011; (**d**, paxilline) n=10 from three mice, ****P*=0.0001; (**d**, *S*-ketamine, 10 μM) n=5 from two mice, ****P*=0.0001; (**d**, *S*-ketamine, 1 μM) n=5 from two mice, ****P*=0.0019.

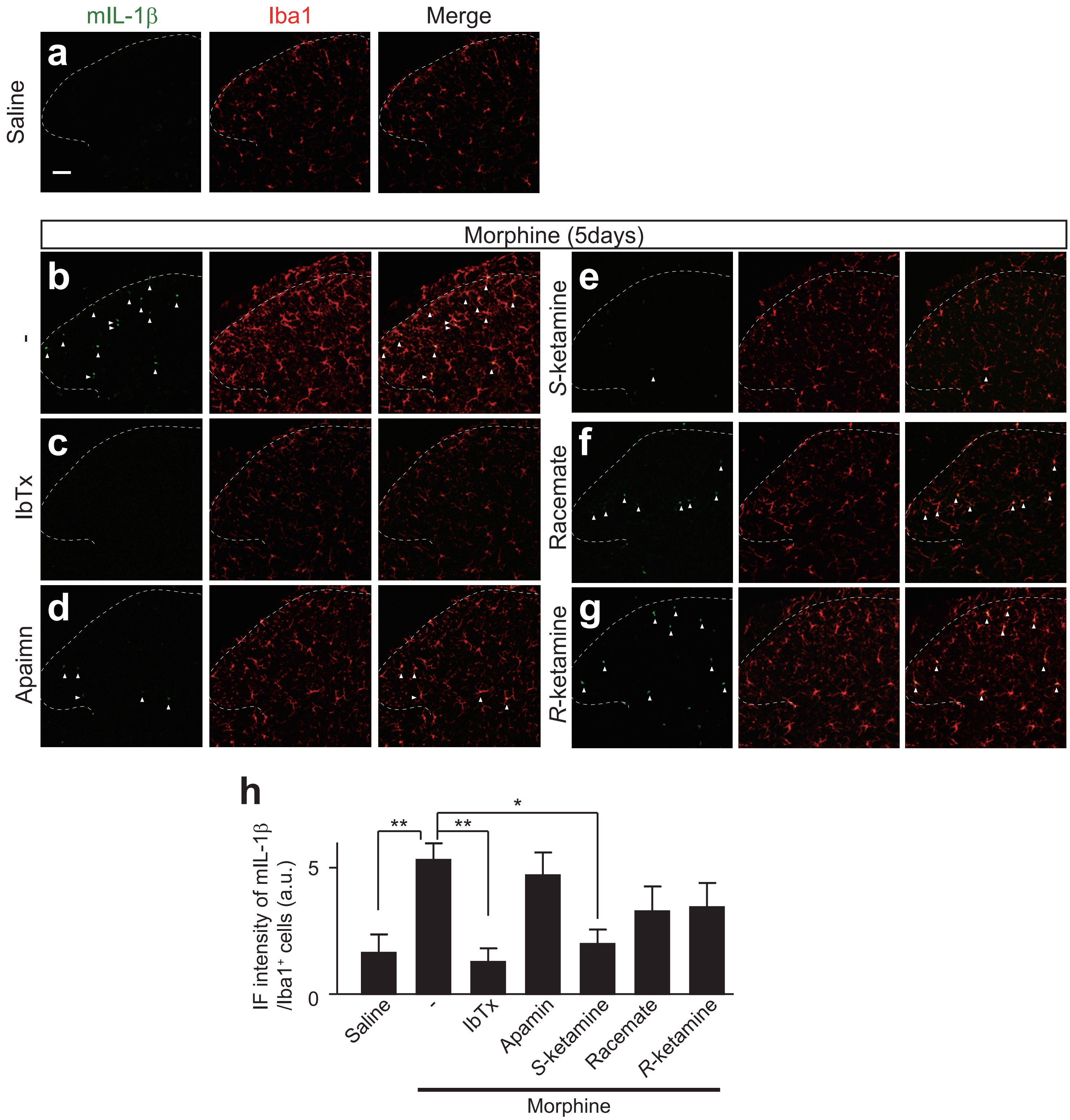

Supplementary Figure 21. The effects of paxilline or S-ketamine on the production of mature interleukin-1β (mIL-1β) following the administration of morphine. (a-g) Double immunofluorescence staining using mIL-1β (green) and Iba1 (red), a marker for microglia, in the L4 spinal dorsal horn. The intrathecal injection of (**c**) IbTx (1 pmol) or apamin (1 pmol) or (**e**) the intraperitoneal injection of *S*-ketamine (2 mg kg^{-1}), (**f**)

racemate (2 mg kg^{-1}), or (**g**) *R*-ketamine (2 mg kg^{-1}) were performed 30 min prior to the administration of morphine (10 mg kg^{-1}). Immunostaining was performed after the 5-day administration of morphine and each of the drugs. The arrowheads indicate mIL-1β immunofluorescence that merged with Iba1 immunofluorescence. Scale bar, 50 μm. (**h**) The average of the immunofluorescence (IF) intensity of mIL-1β within the Iba1-positive cells (n=3-4 section from three-four mice). The data represent the means ± SEM. ***P*=0.0082 (Saline versus Morphine), ***P*=0.0051 (Morphine versus Morhine+IbTx), **P*=0.0352 (Morphine versus Morphine+*S*-ketamine), $F_{6, 47}$=4.628, a one-way ANOVA followed by Tukey' s *post hoc* test).

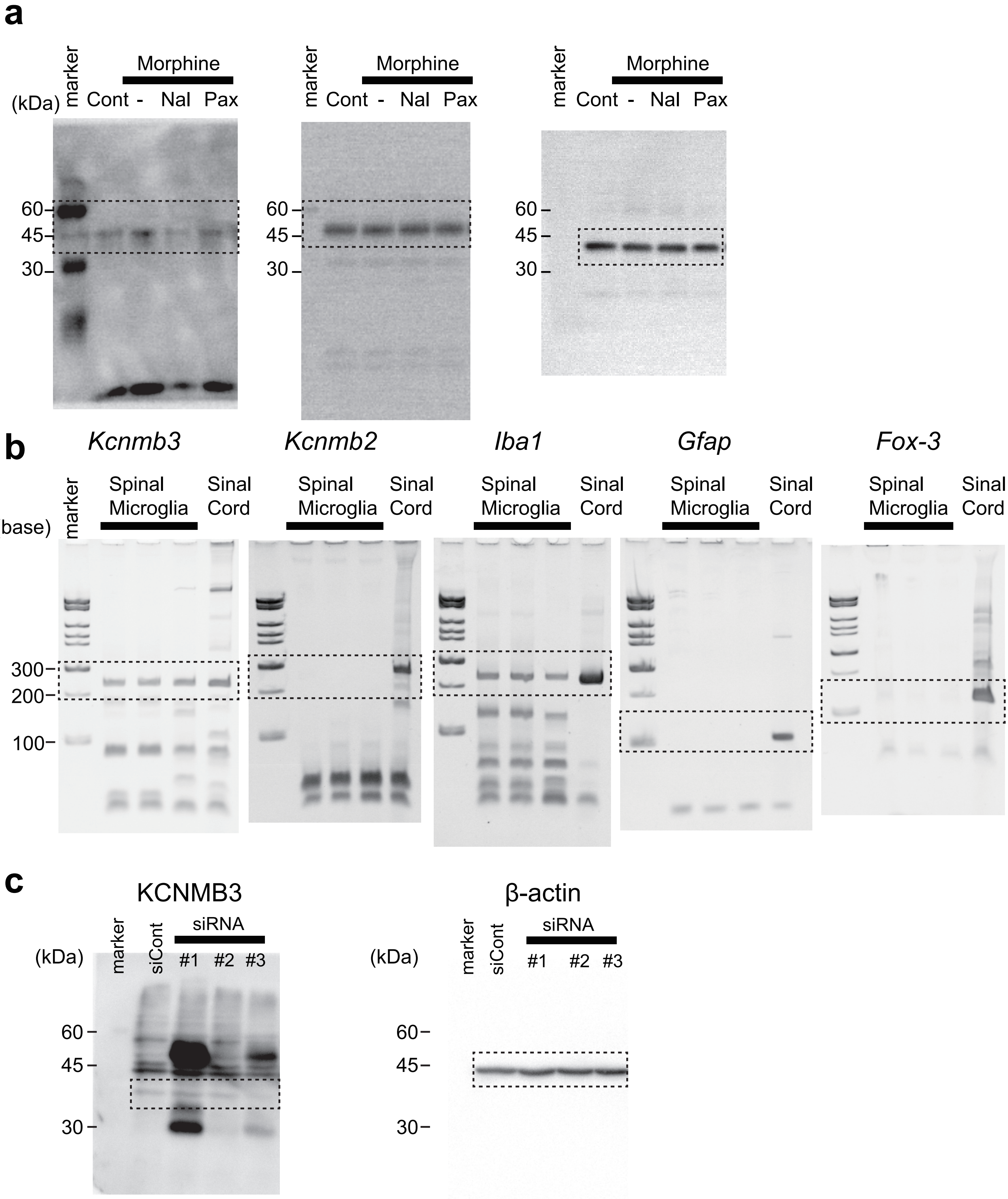

Supplementary Figure 22. Full-length images of blots and gels presented in Figures 5 and 7. (**a**) Figure 5d. (**b**) Figure 7a. (**c**) Figure 7c. Rectangles indicate the regions used in the figures.

Supplementary Table 1. Primer seaquences

Gene	Direction	Primer sequence
Mouse *Kcnma1*	Forward<br>Reverse	5’-CAACAAGGCCCATCTGCTCA<br>5’-GGCAGACTTGTACTCAATGGC
Mouse *Kcnmb1*	Forward<br>Reverse	5’-GTTTTTATCCCGGGGCAAGC-3’<br>5’-CCGCCAAGATGGATAGGGAC-3’
Mouse *Kcnmb2*	Forward<br>Reverse	5’-AGTACACTCCGGTGTCTGGA-3’<br>5’-CTTCTTCTGTCCACACGCTCT-3’
Mouse *Kcnmb3*	Forward<br>Reverse	5’-TCAAACTGCACCACTGTCCA-3’<br>5’-GAGCAGATCATCCCGGTCTC-3’
Mouse *Kcnmb4*	Forward<br>Reverse	5’-GATGGCGAAGCTCAGGGTGTCT-3’<br>5’-CTCCTCCCCGTTAAGAGAACT-3’
Rat *Kcnma1*	Forward<br>Reverse	5’-TGGCTTGCGGTTTATTGCAG-3’<br>5’-GCGTCATCACCCTCTTTCCA-3’
Rat *Kcnmb1*	Forward<br>Reverse	5’-ACCCAGGGAACTAAATGACTGT-3’<br>5’-GGATTTGGGGCCCATAGAGG-3’
Rat *Kcnmb2*	Forward<br>Reverse	5’-TATATGGACCAGTGGCCGGA-3’<br>5’-GGCTTCTTCTGTCCATACACTCT-3’
Rat *Kcnmb3*	Forward<br>Reverse	5’-TCAAACTGCACCACTGTCCA-3’<br>5’-GAGCAGATCATCCCGGTCTC-3’
Rat *Kcnmb4*	Forward<br>Reverse	5’-GACTAACCCCAAGTGCTCCT-3’<br>5’-AGCACACGGGATACAAGCAA-3’
Gapdh	Forward<br>Reverse	5’-AGGTCGGTGTGAACGGATTTG-3’<br>5’-TGTAGACCATGTAGTTGAGGTCA-3’
Iba1	Forward<br>Reverse	5’-GATTTGCAGGGAGGAAAAGCT-3’<br>5’-AACCCCAAGTTTCTCCAGCAT-3’
Gfap	Forward<br>Reverse	5’-GAGTGGTATCGGTCTAAGTTTGCA-3’<br>5’-GCGGCGATAGTCGTTAGCTT-3’
Fox-3	Forward<br>Reverse	5’-CCAGGCACTGAGGCCAGCACACAGC-3’<br>5’-CTCCGTGGGGTCGGAAGGGTGG-3’
